# Early Life Antibiotics Influence In Vivo and In Vitro Mouse Intestinal Epithelium Maturation and Functioning

**DOI:** 10.1016/j.jcmgh.2021.05.019

**Published:** 2021-06-06

**Authors:** Tânia Martins Garcia, Manon van Roest, Jacqueline L.M. Vermeulen, Sander Meisner, Wouter L. Smit, Joana Silva, Pim J. Koelink, Jan Koster, William J. Faller, Manon E. Wildenberg, Ruurd M. van Elburg, Vanesa Muncan, Ingrid B. Renes

**Affiliations:** 1 Department of Gastroenterology and Hepatology, Tytgat Institute for Intestinal and Liver Research, Amsterdam Gastroenterology Endocrinology Metabolism, Amsterdam, the Netherlands; 4 Department of Oncogenomics, Amsterdam, the Netherlands; 5 Department of Pediatrics, Amsterdam University Medical Center, University of Amsterdam, Amsterdam, the Netherlands; 2 Department of Medical Microbiology, University Medical Center Utrecht, Utrecht, the Netherlands; 3 Department of Oncogenomics, Netherlands Cancer Institute, Amsterdam, the Netherlands; 6 Danone Nutricia Research, Utrecht, the Netherlands

**Keywords:** Antibiotic Treatment, Neonatal Intestine, Fetal Organoids, AB, antibiotics, ACS, apical canalicular system, Arg2, arginase 2, Ass1, argininosuccinate synthase 1, ATP, adenosine triphosphate, EEC, enteroendocrine cell, ECAR, extracellular acidification rate, EpCAM, epithelial cell adhesion molecule, FCCP, carbonyl cyanide-4 (trifluoromethoxy) phenylhydrazone, FITC, fluorescein isothiocyanate, GIP, gastric inhibitory polypeptide, GO, Gene Ontology, GSEA, gene set enrichment analysis, IEC, intestinal epithelial cell, NEC, necrotizing enterocolitis, OCR, oxygen consumption rate, P, postnatal day, PBS, phosphate-buffered saline, qRT-PCR, quantitative reverse-transcription polymerase chain reaction, SI, small intestine, Sis, sucrase-isomaltase, 2-DG, 2-deoxy-glucose

## Abstract

**Background & Aims:**

The use of antibiotics (ABs) is a common practice during the first months of life. ABs can perturb the intestinal microbiota, indirectly influencing the intestinal epithelial cells (IECs), but can also directly affect IECs independent of the microbiota. Previous studies have focused mostly on the impact of AB treatment during adulthood. However, the difference between the adult and neonatal intestine warrants careful investigation of AB effects in early life.

**Methods:**

Neonatal mice were treated with a combination of amoxicillin, vancomycin, and metronidazole from postnatal day 10 to 20. Intestinal permeability and whole-intestine gene and protein expression were analyzed. IECs were sorted by a fluorescence-activated cell sorter and their genome-wide gene expression was analyzed. Mouse fetal intestinal organoids were treated with the same AB combination and their gene and protein expression and metabolic capacity were determined.

**Results:**

We found that in vivo treatment of neonatal mice led to decreased intestinal permeability and a reduced number of specialized vacuolated cells, characteristic of the neonatal period and necessary for absorption of milk macromolecules. In addition, the expression of genes typically present in the neonatal intestinal epithelium was lower, whereas the adult gene expression signature was higher. Moreover, we found altered epithelial defense and transepithelial-sensing capacity. In vitro treatment of intestinal fetal organoids with AB showed that part of the consequences observed in vivo is a result of the direct action of the ABs on IECs. Lastly, ABs reduced the metabolic capacity of intestinal fetal organoids.

**Conclusions:**

Our results show that early life AB treatment induces direct and indirect effects on IECs, influencing their maturation and functioning.

## Graphical abstract

**Figure:**
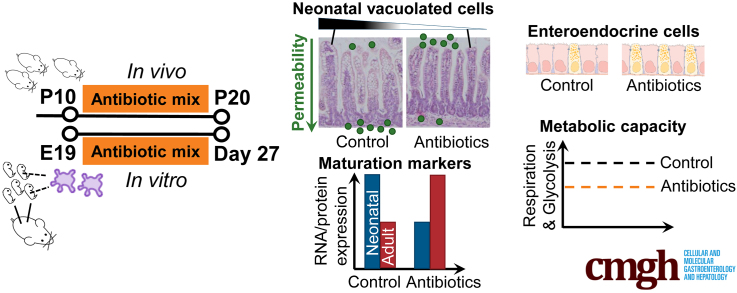

**Summary**

Early life antibiotics decrease intestinal permeability, accelerate epithelial maturation, induce enteroendocrine cells, and limit the metabolic capacity of intestinal epithelial cells. In part, these effects are consequence of the direct action of antibiotics on epithelial cells, independent of intestinal microbiota disturbances.

Infectious diseases are one of the leading causes of mortality in children younger than the age of 5 years. Because antibiotics (ABs) are the cornerstone of adequate treatment of bacterial infections, it is not surprising that ABs are the most prescribed drugs in early childhood.^1^, ^2^, ^3^ Often, ABs of different classes are used in combination to treat suspected or confirmed infections caused by different pathogens. Three frequently used ABs in children are amoxicillin, metronidazole, and vancomycin.^1^^,^^3^, ^4^, ^5^, ^6^, ^7^, ^8^, ^9^, ^10^, ^11^ Amoxicillin is prescribed when there is suspicion of systemic infection, affecting both gram-positive and gram-negative bacteria.^8^^,^^12^, ^13^, ^14^ When gastrointestinal complications develop, caused by anaerobic bacteria, metronidazole is given, frequently in combination with amoxicillin.^11^^,^^15^, ^16^, ^17^, ^18^ Upon onset of systemic or gastrointestinal infections with gram-positive bacteria, especially in the case of *Staphylococcus aureus* and *Clostridium difficile*, vancomycin is administered.^5^^,^^8^^,^^11^^,^^19^, ^20^, ^21^, ^22^ The combined use of the earlier-mentioned ABs is also common.^8^^,^^11^^,^^17^^,^^20^, ^21^, ^22^ Although ABs have a crucial and beneficial role in treating bacterial infections, they also have several short- and long-term detrimental effects. Use of ABs in early life is associated with necrotizing enterocolitis (NEC),^23^, ^24^, ^25^ infantile colics,^26^^,^^27^ and eczema. Later in life, diseases such as allergy,^28^, ^29^, ^30^ obesity,^31^, ^32^, ^33^, ^34^ and inflammatory bowel diseases^35^, ^36^, ^37^, ^38^ have also been linked with prolonged AB exposure early in life. However, how ABs can affect host cells and therefore contribute to disease development still is not clear.

Increased susceptibility to diseases after AB treatment can be a result of both indirect and direct effects on host cells. ABs disturb the microbial community, which indirectly affects gut homeostasis and perturbs the function of intestinal epithelial cells (IECs).^35^^,^^39^, ^40^, ^41^, ^42^, ^43^, ^44^, ^45^, ^46^, ^47^ When microbiota disturbance occurs during a specific neonatal time window, the development of the gut immune system is compromised, leaving the organism more sensitive to immune pathologies later in life.^30^^,^^48^, ^49^, ^50^, ^51^, ^52^, ^53^, ^54^ At the same time, ABs can directly influence IECs, independent of the microbiota.^55^, ^56^, ^57^ It has been shown that ABs directly elicit various immunomodulatory effects.^58^ Furthermore, it was found that prolonged treatment of human fibroblasts with specific antibiotics affects mitochondrial respiration.^59^ More recently, it was shown that one third of the AB-induced changes in host intestinal epithelial gene expression could be attributed to direct regulation of their expression by the ABs, and not by a shift to a different microbiota composition.^57^

In previous studies, the impact of ABs on the intestine and the intestinal epithelium was investigated in adult mice.^39^, ^40^, ^41^^,^^44^, ^45^, ^46^^,^^57^ However, the effects of ABs in adult mice likely are not the same as in neonatal mice because the gut of neonatal mice has not gained its full function and still is maturing up until weaning, including the suckling-to-weaning transition.^60^, ^61^, ^62^ Intestinal maturation in vivo has been proposed to occur in a wave from proximal to distal intestinal regions.^63^ In mice, this process takes place from postnatal day (P) 14 to P28, and prepares the intestine for the switch to a solid diet, resulting in several changes in epithelial cell functions.^61^ The immature intestinal epithelium is characterized by the presence of an apical canalicular system (ACS) on the apical side of the absorptive enterocytes.^64^, ^65^, ^66^, ^67^ ACSs are responsible for the active endocytosis of maternal immunoglobulins and macromolecules present in milk and feed into supranuclear vacuoles, which are most abundant in the distal small intestine.^68^^,^^69^ The presence of ACSs contributes to the increased intestinal permeability that is characteristic of the neonatal period and gradually decreases as a result of the replacement of cells containing ACSs with adult enterocytes.^68^^,^^70^ Other markers of the immature intestine are the neonatal fragment crystallizable receptor (FcRn), which mediates the uptake of IgG from breast milk,^71^^,^^72^ and argininosuccinate synthase 1 (Ass1), the rate-limiting enzyme in arginine biosynthesis, a semi-essential amino acid that is not present in milk.^73^ During the suckling-to-weaning transition, vacuolated enterocytes disappear, the intestinal epithelium tightens, and its permeability becomes more selective.^72^ The mentioned neonatal markers decrease in expression, while adult brush-border enzymes sucrase-isomaltase (Sis)^74^ and arginase 2 (Arg2)^75^ start to be expressed to digest solid food. The emerging Paneth cells produce lysozyme-1 (Lyz1) and Reg-3 lectins,^76^, ^77^, ^78^, ^79^ and by 1 month of age the intestine is fully matured and has achieved all adult characteristics. Recently, our group developed an in vitro model to study gut neonatal development using a prolonged culture of fetal mice gut organoids. We established that fetal organoids isolated from the intestine of mice at late fetal stage (embryonic day 19) undergo intrinsic maturation in vitro, recapitulate suckling-to-weaning transition, and that extrinsic factors can be applied to the culture to investigate whether they can modulate intestinal epithelial maturation.^80^^,^^81^

Here, we combined 2 different approaches to study how ABs affect the neonatal intestinal epithelium. First, we investigated the effect of early life ABs on neonatal IECs in mice, either by indirect or direct mechanisms, and, second, we determined the direct effect of ABs on IECs in organoids.

## Results

### Mice Pups Treated With Early Life Antibiotics Show Decreased Intestinal Permeability and Loss of Vacuolated Enterocytes

**Figure 1:**
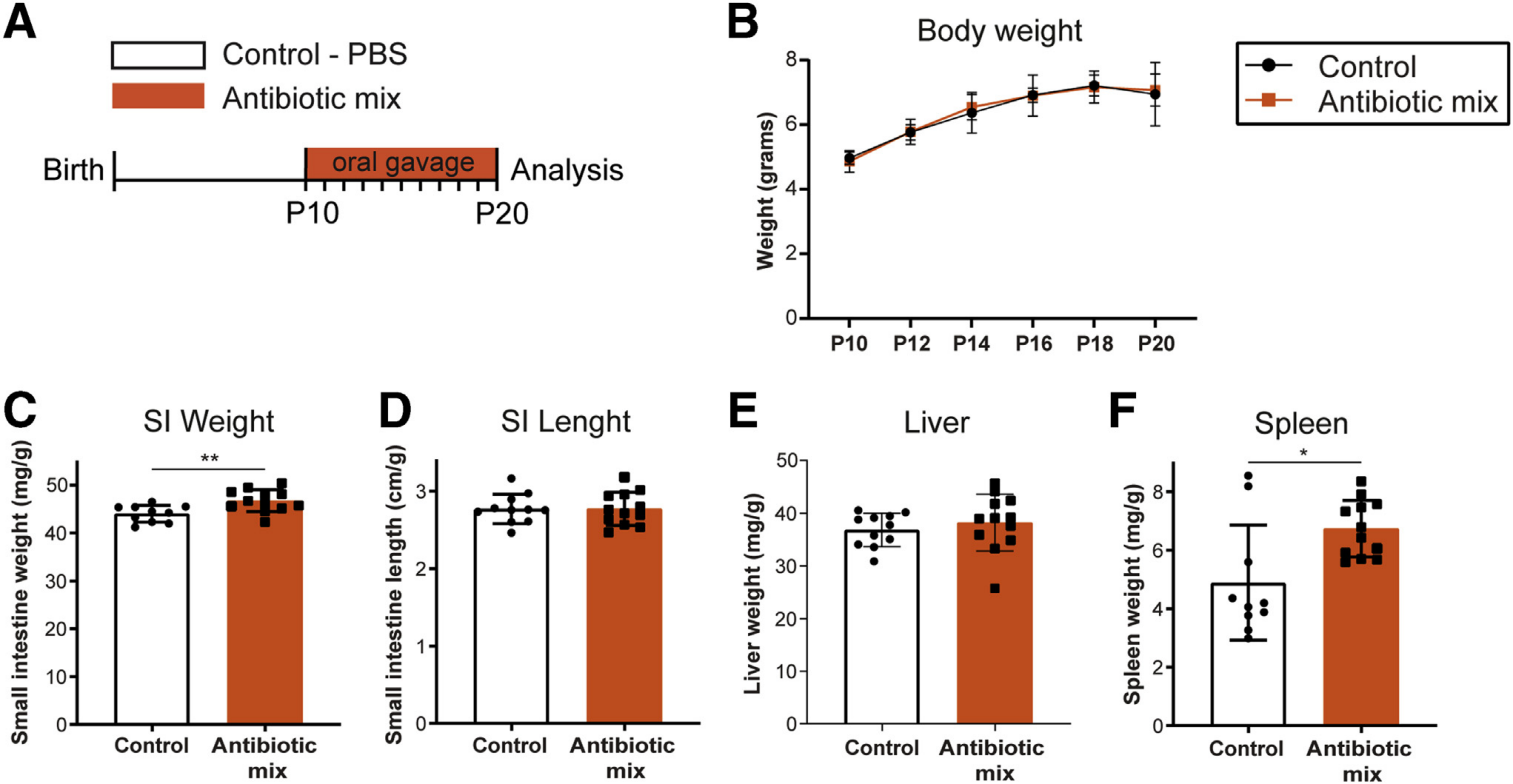
**In vivo growth and macroscopic assessment of small intestine, liver, and spleen.** (*A*) Experimental design of in vivo antibiotic treatment of pups between P10 and P20. Antibiotic mix: amoxicillin, metronidazole, and vancomycin. All analyses were performed at P20. (*B*) Weight of pups was measured every 2 days during antibiotic mix treatment between P10 and P20. (*C*) Small intestine weight, relative to body weight. (*D*) Small intestine length, relative to body weight. (*E*) Liver weight, relative to body weight. (*F*) Spleen weight, relative to body weight. Statistical analysis was performed by a (*B*) 2-way analysis of variance test with the Sidak multiple comparisons test or a (*C–F*) 2-tailed unpaired *t* test. Error bars indicate means ± SD. Levels of significance are indicated: ∗*P* < .05, ∗∗*P* < .01. *n* = 9–12 pups per group.

To investigate the effects of ABs during neonatal development in vivo, we treated mice with ABs as of P10 because this time point of murine intestinal development corresponds to the newborn human intestine.^82^ P10 mice were treated daily and orally for 10 days with the combination of 3 frequently used ABs in neonates and children: amoxicillin (β-lactam), vancomycin (glycopeptide), and metronidazole (nitroimidazole)^1^^,^^3^^,^^5^^,^^6^^,^^8^^,^^10^^,^^83^ (Figure 1*A*). During this period, weight gain was similar in AB-treated and control pups (Figure 1*B*), indicating that the AB treatment did not affect growth. At P20, the small intestine was significantly heavier in the AB-treated pups compared with control pups, although the intestinal length was similar in both groups (Figure 1*C* and *D*). The liver weight was similar between both groups, but spleens of AB-treated pups were significantly heavier, which could indicate an inflammatory process (Figure 1*E* and *F*).

**Figure 2:**
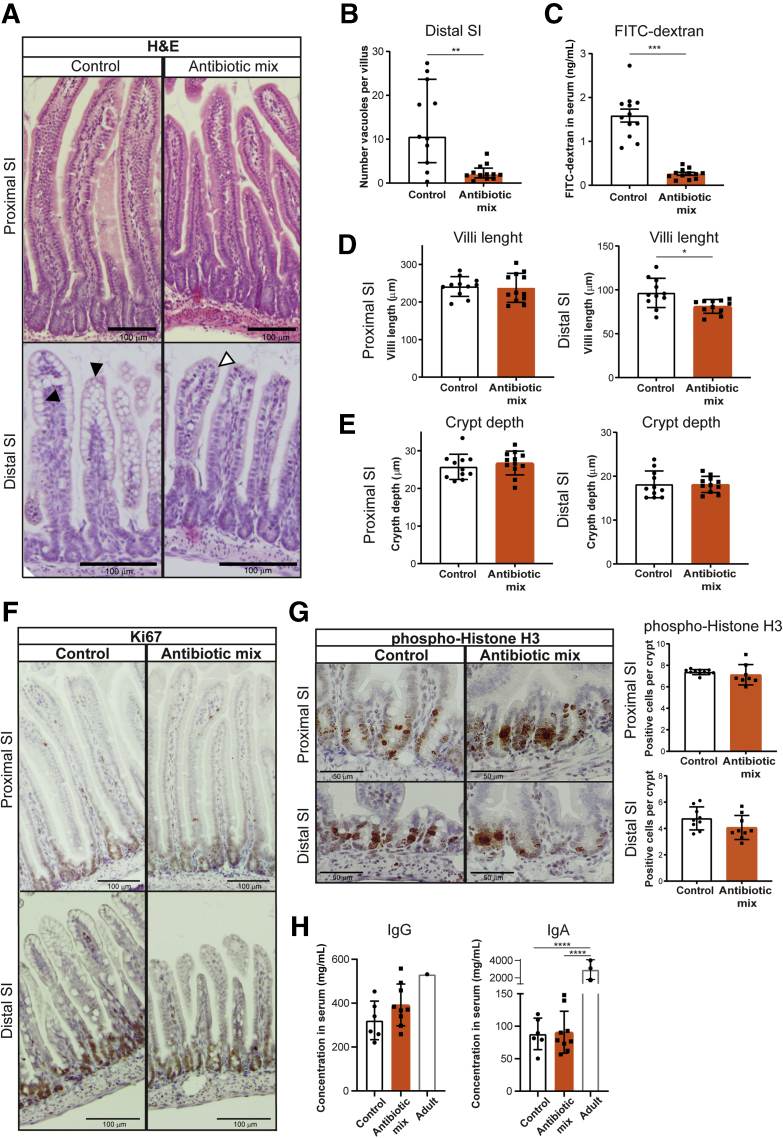
**Early life antibiotics affect intestinal barrier function in vivo, particularly in the distal small intestine.** (*A*) H&E staining of proximal and distal small intestine. *Black triangles* indicate vacuolated enterocytes and *white triangles* indicate nonvacuolated enterocytes. *Scale bars*: 100 μm. (*B*) Quantification of the number of vacuoles per villi in the distal small intestine. (*C*) Permeability assay assessed by FITC-dextran concentration in serum 4 hours after oral gavage. (*D*) Villi length in proximal and distal small intestine. (*E*) Crypt depth in proximal and distal small intestine. Immunohistochemistry of (*F*) proliferation markers Ki67 and (*G*) phosphorylated histone H3 in proximal and distal small intestine. *Scale bars*: 100 μm. (*H*) Serum concentration of IgG and IgA in control and antibiotic mix–treated pups at P20 compared with adult mice. Statistical analysis was performed by the Mann–Whitney test because data were not normally distributed when assessed by the (*B*) D’Agostino and Pearson normality test, the (*C–E* and *G*) 2-tailed unpaired *t* test or (*H*) 1-way analysis of variance with the Tukey multiple comparisons test. Error bars indicate (*B*) medians with interquartile range or (*C–E*, *G*, and *H*) means ± SEM. Levels of significance are indicated: ∗*P* < .05, ∗∗*P* < .01, ∗∗∗*P* < .0001, ∗∗∗∗*P* < .0001. (*B–E* and *G*) *n* = 9–12 pups per group and (*H*) *n* = 6–9 P20 samples per group and *n* = 1–3 adult samples.

Because the small intestine (SI) follows a maturation wave along the proximal-to-distal axis,^63^ which displays distinct functional and genetic profiles^84^, ^85^, ^86^ that can be affected differently by AB treatment,^87^ we analyzed the proximal and distal parts of the SI separately. The histology of the SI showed no major differences in overall morphology (Figure 2*A*). However, we observed a strong reduction in the number of vacuolated enterocytes containing ACSs in the distal SI after AB treatment (Figure 2*A* and *B*). Intestinal permeability as measured by the passage of fluorescein isothiocyanate (FITC) dextran through the intestinal epithelial barrier was significantly lower in AB-treated pups compared with control pups (Figure 2*C*). Although the length of the villi was similar in the proximal SI, villi in the distal SI were shorter in AB-treated pups compared with control pups (Figure 2*D*). However, crypt depth in proximal and distal SI was not affected by AB treatment (Figure 2*E*). Cellular proliferation was similar between the 2 groups, in both proximal and distal SI, as shown by immunodetection of the proliferation marker Ki67 (Figure 2*F*), although the mitotic marker phosphorylated histone H3 followed a trend toward a lower number of proliferating cells in the distal SI (Figure 2*G*). Because we observed a strong reduction in both ACS and intestinal permeability, we examined whether AB exposure decreased the transfer of immunoglobulins through the neonatal intestinal epithelium. Serum IgG and IgA concentrations as measured by enzyme-linked immunosorbent assay were similar between the 2 groups at P20 (Figure 2*H*). The decreased number of vacuolated enterocytes and reduced intestinal permeability suggest an effect of early life ABs on the maturation of the intestinal barrier.

### Early Life Antibiotics Induce Gene Expression Changes in Small Intestine Epithelial Cells In Vivo

**Figure 3:**
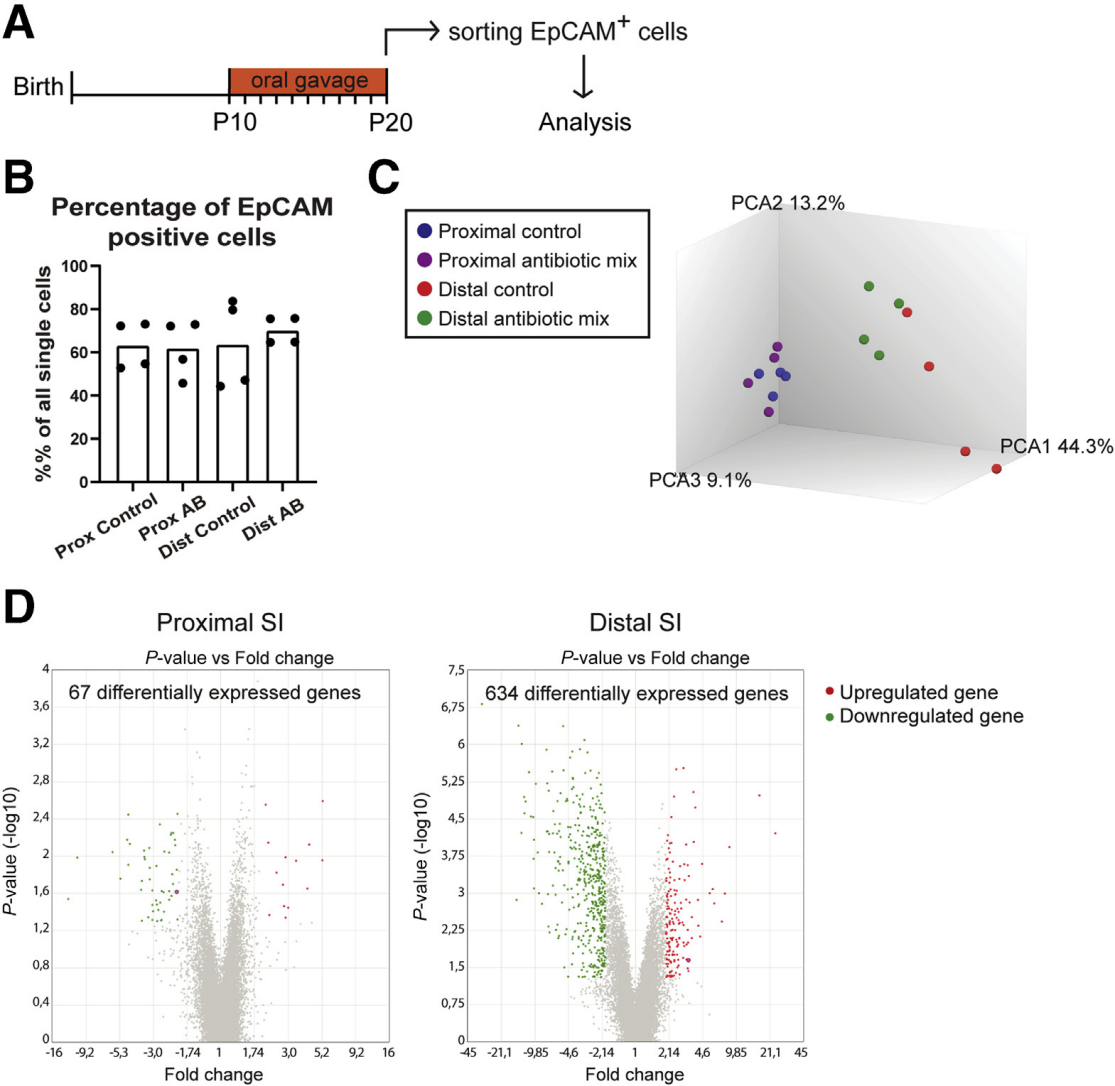
**Genome-wide gene expression analysis of sorted intestinal epithelial cells.** (*A*) Experimental design of genome-wide gene expression analysis of fluorescence-activated cell sorter (FACS)-sorted P20 intestinal epithelial cells. (*B*) Percentage of EpCAM stained cells in the total number of FACS-sorted intestinal epithelial cells per condition. (*C*) Principal component analysis (PCA) of sorted epithelial cells from P20 proximal and distal small intestine treated with the antibiotic mix or PBS (control). (*D*) Volcano plots of microarray analysis showing genes differentially expressed between control and antibiotic-treated FACS-sorted P20 intestinal epithelial cells. *Green dots* identify down-regulated genes and *red dots* identify up-regulated genes. Statistical analysis by empirical Bayes analysis of variance, *P* < .05 cut-off. *n* = 4 samples per group.

Decreased intestinal barrier and disappearance of ACS are the prime characteristics of the intestinal maturation process occurring during the replacement of neonatal epithelium by adult epithelium. To determine the effect of the AB treatment on intestinal epithelial maturation, we performed genome-wide gene expression analysis on messenger RNA from proximal and distal SI epithelial cells (Figure 3*A*). The epithelial cell adhesion molecule (EpCAM) marker was used to isolate the epithelial cells of both regions because no difference in the percentage of this marker was detected between control and antibiotic-treated samples (Figure 3*B*). Principal component analysis showed a clear separation between proximal and distal SI epithelial cells along with the first component (Principal Component Analysis 1 (PCA1) 44.3%) (Figure 3*C*). Despite some variance between samples, AB-treated epithelial cells separated from control epithelial cells along the Principal Component Analysis 2 (PCA2) (13.2%). This separation between AB and control groups was more evident in the epithelial cells of the distal SI compared with the proximal SI (Figure 3*C*). Indeed, the differential gene expression analysis showed 67 genes in the proximal SI and 634 genes in the distal SI epithelial cells that were up-regulated or down-regulated 2-fold or more in the samples treated with early life ABs (Figure 3*D*, Supplementary Tables 1 and 2).

**Figure 4:**
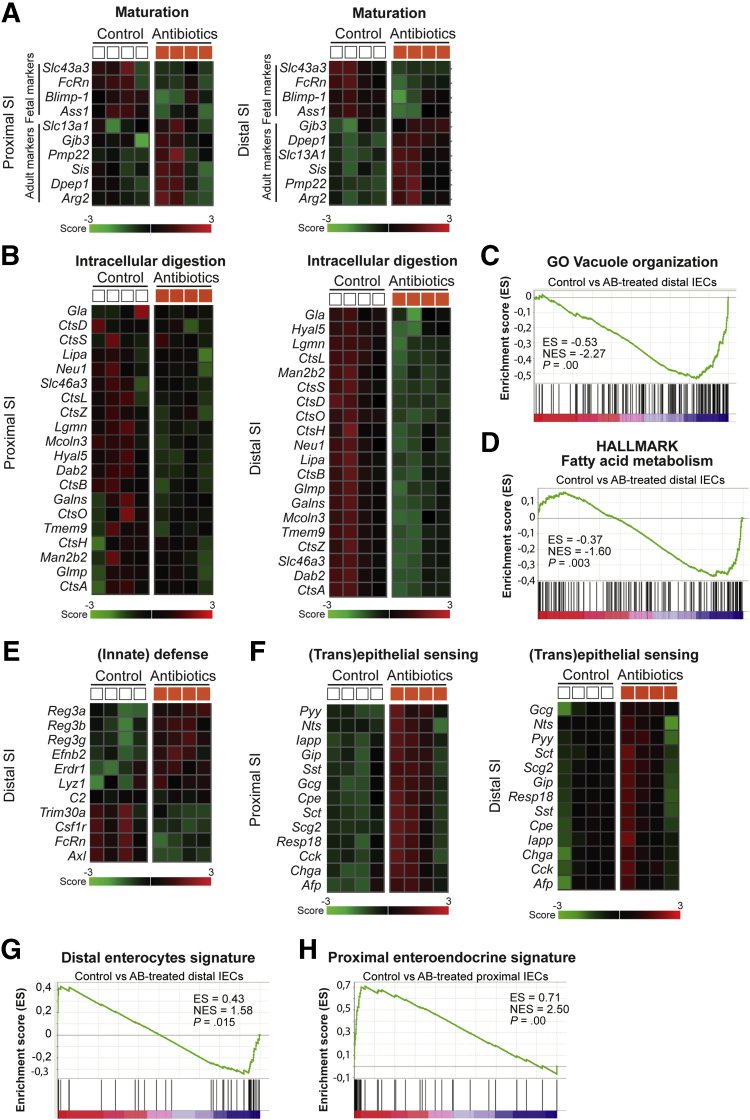
**Differential gene expression analysis and GSEA of sorted intestinal epithelial cells after antibiotic treatment.** Curated heatmaps of selected genes from top down-regulated and top up-regulated genes, based on biological interest and grouped according to function (*A*) maturation and (*B*) intracellular digestion in proximal and distal epithelial cells. The *colored bar* represents the expression level from low (green) to high (red). GSEA plots comparing control and antibiotic-treated distal SI epithelial cells against gene sets of (*C*) GO vacuole organization and (*D*) HALLMARK fatty acid metabolism. Enrichment score (ES), normalized enrichment score (NES), and *P* values are indicated in the image. Curated heatmaps of selected genes from top down-regulated and top up-regulated genes, based on biological interest and grouped according to function (*E*) (innate) defense in distal epithelial cells and (*F*) (trans)epithelial sensing in proximal and distal epithelial cells. The *colored bar* represents the expression level from low (green) to high (red). GSEA plots comparing (*G*) distal SI control and antibiotic-treated epithelial cells against a published gene set of distal SI enterocytes and (*H*) comparing proximal SI control and antibiotic-treated epithelial cells against a proximal SI enteroendocrine cell signature. ES, NES, and *P* values are indicated in the image. Statistical analysis by empirical Bayes analysis of variance, *P* < .05 cut-off. *n* = 4 samples per group.

Within the sets of the most differentially expressed genes, many of the known fetal/neonatal and adult maturation markers were changed in the distal SI (Figure 4*A*). Specifically, fetal/neonatal markers *FcRn*, *Blimp-1*, *Ass1*, and *Slc43a3* were down-regulated after AB treatment and adult markers *Sis*, *Arg2*, *Dpep1*, *Pmp22*, *Gjb3*, and *Slc13a1* were up-regulated in epithelial cells of AB-treated pups (Figure 4*A*). These differences were less obvious in the proximal SI (Figure 4*A*). In addition, in the distal SI, intracellular digestion genes, that is, genes involved in the formation of the lytic supranuclear vacuoles that compose the ACS (*Slc46a3I*, *Tmem9*, *Dab2*, *Mcoln3*, and *Glmp*), as well as genes related to the degradation of milk macromolecules within these vacuoles (*Hyal5*, several *Cts* genes, *Galns*, *Neu1*, *Lgmn*, *Lipa*, *Man2b2*), were down-regulated significantly in AB-treated pups (Figure 4*B*). The differences in intracellular digestion genes were less evident in proximal SI epithelial cells (Figure 4*B*). Accordingly, gene set enrichment analysis (GSEA) using sets of genes from the Molecular Signatures Database, such as hallmark gene set collection (HALLMARK) and Gene Ontology (GO), showed HALLMARK vacuole organization and GO fatty acid metabolism gene sets enriched in distal SI epithelial cells isolated from control pups (Figure 4*C* and *D*). Furthermore, changes in (innate) defense genes also were observed in the distal SI after AB treatment, such as up-regulation of *Lyz1* and Reg-3 lectins (Figure 4*E*). AB-treated pups also showed lipid, short-chain fatty acids, and bile acid metabolism in distal SI epithelial cells being affected by early life AB: lipid transporter *Apoa4*; cholesterol transporters *Abcg8* and *Npc1l1*; lipid metabolism enzymes *Acot5*, *Acot12*, *Acadl*, *Acsl1*, and *Fabp6*; and short-chain fatty acid mitochondrial enzyme *Acss1* were down-regulated and primary bile acid uptake transporter *AsbtI*/*Slc10a2* was up-regulated (Supplementary Table 2). Moreover, distal SI epithelial cells of pups treated with AB showed down-regulation of genes encoding subunits of the mitochondrial complex IV (*Cox6b2*) and complex V (*Atp5e*), as well as down-regulation of *Slc2a2*/*Glut2* and *Slc37a4*, and up-regulation of *Slc2a1/Glut1*, all glucose transporters (Supplementary Table 2).

Of the differentially expressed genes in the proximal SI epithelial cells, the top up-regulated genes were markers of enteroendocrine cells (Figure 4*F*). This secretory cell type functions as (trans)epithelial sensors within the gut to regulate energy homeostasis by producing hormones, such as neurotensin (Nts), somatostatin (Sst), secreting (Sct), gastric inhibitory polypeptide (Gip), cholecystokinin (Cck), glucagon (Gcg), peptide YYY *(*Pyy), and chromogranin A (ChgA). Although the fold-change of enteroendocrine cell (EEC) markers was lower in the distal SI epithelial cells, their expression also was up-regulated significantly after AB treatment (Figure 4*F*). In addition, comparison of our data set with previously published single-cell RNA sequencing gene sets from sorted EpCAM-positive intestinal epithelial cells of adult C57BL/6 wild-type mice (GEO GSE92332)^88^ confirmed that adult enterocyte markers were enriched significantly in distal SI epithelial cells of AB-treated pups compared with control and that adult enteroendocrine cell markers were enriched significantly in proximal SI epithelial cells of AB-treated pups compared with control (Figure 4*G* and *H*).

Together, these data show that ABs in early life causes gene expression changes specifically in the proximal and distal small intestine, which are associated with intestinal epithelial maturation, intracellular digestion, (innate) defense, and (trans)epithelial sensing functions of the intestinal epithelial cells.

### Antibiotic Treatment in Early Life Leads to Precocious Maturation of the Intestinal Epithelium and Increased Expression of Enteroendocrine Cell Markers In Vivo

**Figure 5:**
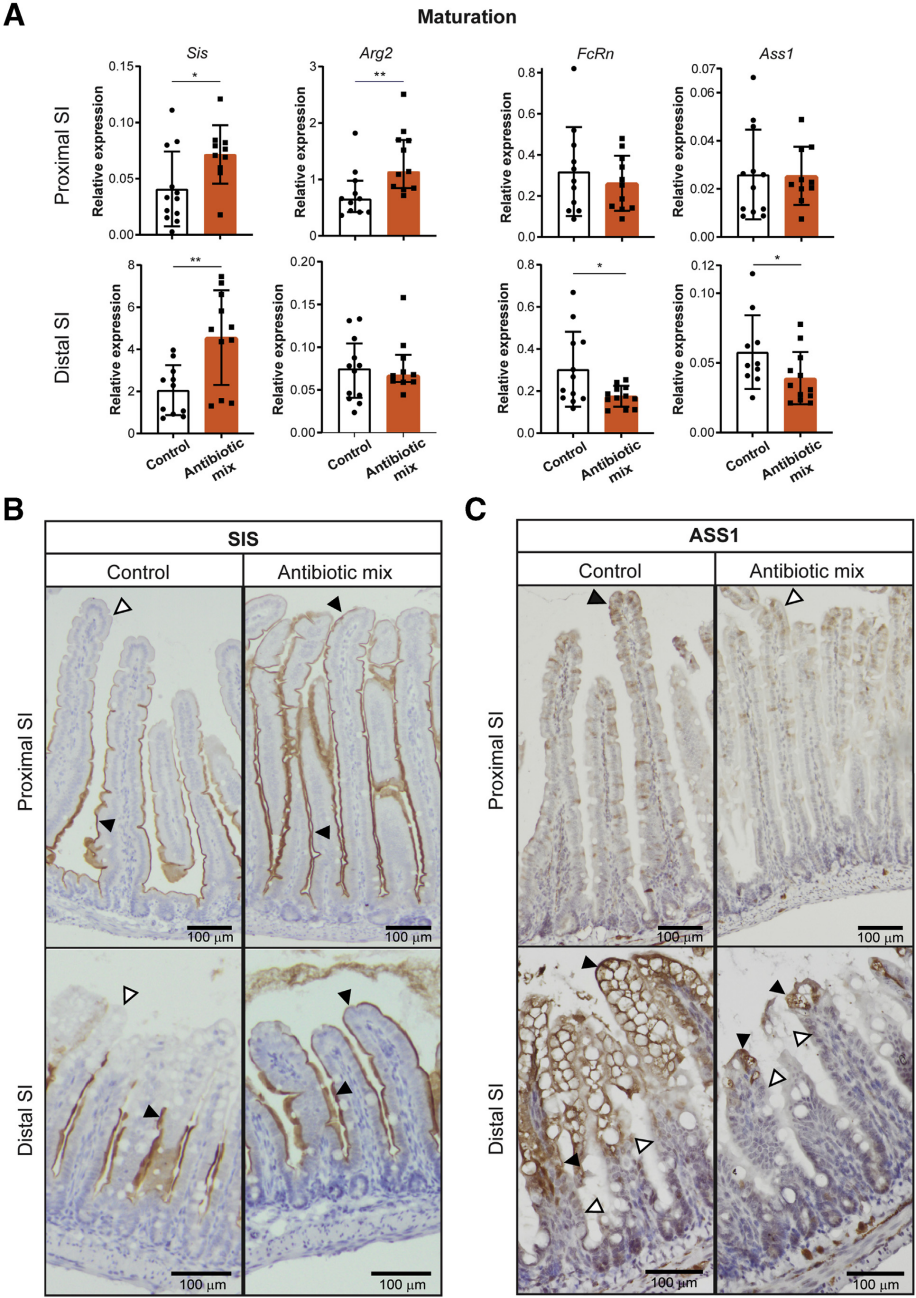
**Early life antibiotics induce in vivo precocious maturation of the intestinal epithelium.** (*A*) Whole-tissue real-time qPCR analysis of adult maturation markers *Sis* and *Arg2* and of fetal maturation markers *FcRn* and *Ass1* in proximal and distal small intestine. Relative expression to reference genes *Cyp* and *Ppib*. Immunohistochemistry of (*B*) adult marker Sis and (*C*) fetal marker Ass1 in proximal and distal small intestine. *Black triangles* indicate positive cells and *white triangles* indicate negative cells. *Scale bars*: 100 μm. Statistical analysis was performed by 1-tailed unpaired *t* test (*Sis*, *FcRn*, and *Ass1*) or Mann–Whitney test because data were not distributed normally when assessed by the D’Agostino and Pearson normality test (*Arg2*). Error bars indicate means ± SD (*Sis*, *FcRn*, and *Ass1*) or medians with interquartile range (*Arg2*). Levels of significance are indicated: ∗*P* < .05, ∗∗*P* < .01. *n* = 10–12 pups per group.

We set out to verify the described changes in global transcription by performing an independent in vivo experiment and subsequent whole-tissue analysis by quantitative reverse-transcription polymerase chain reaction (qRT-PCR). This analysis confirmed the increase in relative expression of adult marker *Sis*, but not of *Arg2*, in the distal SI of AB-treated pups compared with control pups (Figure 5*A*). The relative expression of the neonatal markers *FcRn* and *Ass1* on AB treatment was reduced significantly in distal SI (Figure 5*A*). According to genome-wide gene expression analysis, differences in maturation were most obvious in the distal SI (Figure 4*A*). Still, whole-tissue qRT-PCR analysis showed that *Sis* and *Arg2*, but not *FcRn* and *Ass1*, also were increased significantly in proximal SI after AB treatment (Figure 5*A*). In addition, immunohistochemical analysis showed that the adult marker Sis was detected only at the villus tips in both proximal and distal SI of AB-treated pups, but not in control mice (Figure 5*B*). At the same time, expression of Ass1 protein, a marker for neonatal epithelium, was reduced at the villus tips of AB mice, especially in distal SI (Figure 5*C*).

**Figure 6:**
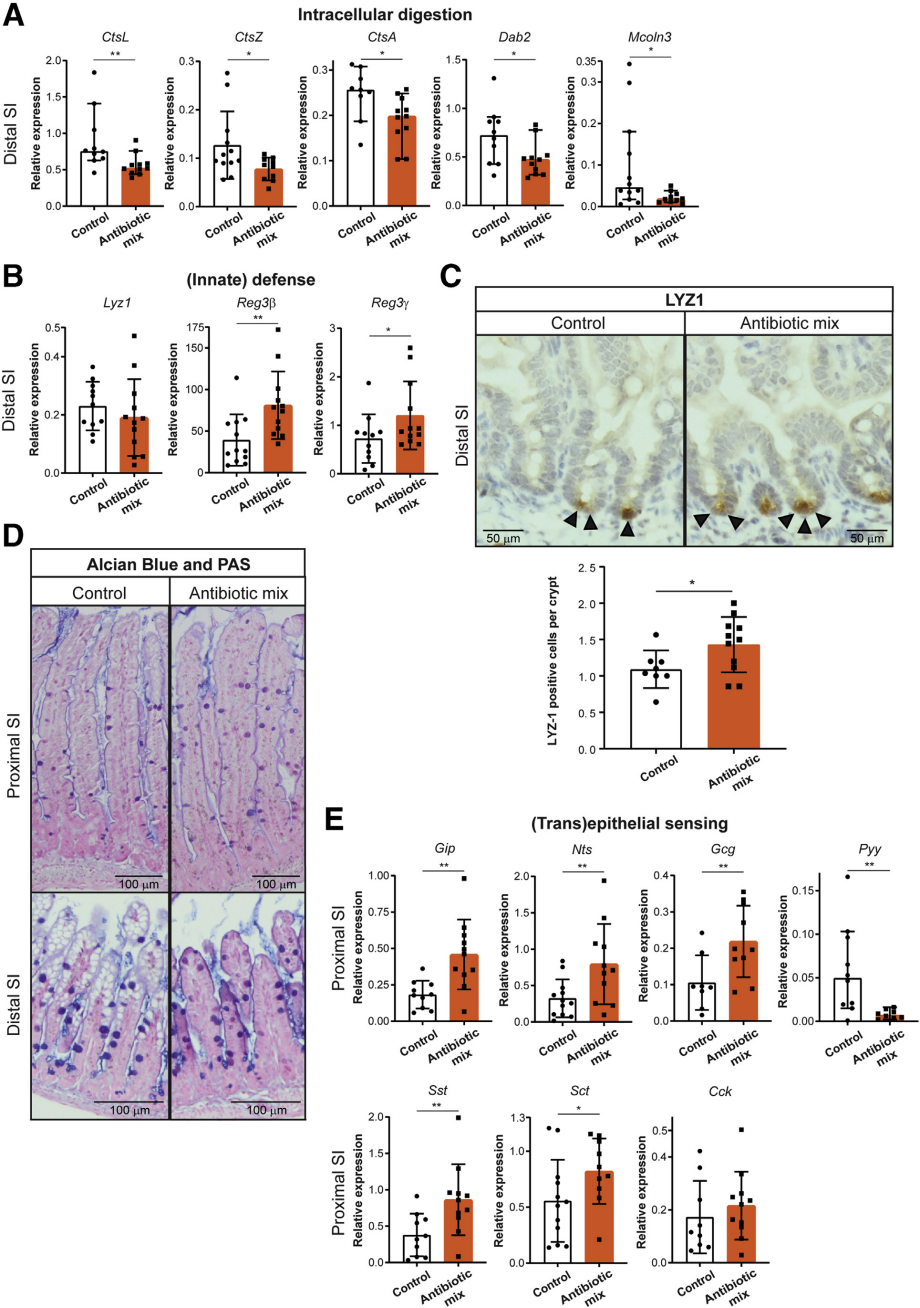
**Treatment with early life antibiotics in vivo decreases expression of intracellular digestion markers and increases expression of innate defense and enteroendocrine cell markers.** Whole-tissue real-time qPCR analysis of (*A*) intracellular digestion markers *CtsL*, *CtsZ*, *CtsA*, *Dab2*, and *Mcoln3* and of (*B*) innate defense markers *Lyz1*, *Reg3β*, and *Reg3ɣ* in distal small intestine. (*C*) Immunohistochemistry of LYZ1 and quantification of amount of lysozyme-1–positive cells per crypt in distal small intestine. *Black arrowheads* indicate positively stained cells. *Scale bars*: 100 μm. (*D*) Alcian blue and periodic acid-Schiff (PAS) staining of proximal and distal small intestine. *Scale bars*: 100 μm. (*E*) Whole-tissue real-time qPCR analysis of (trans)epithelial sensing markers *Gip*, *Nts*, *Gcg*, *Pyy*, *Sst*, *Sct*, and *Cck* in proximal small intestine. Relative expression to reference genes *Cyp* and *Ppib*. Statistical analysis was performed by 1-tailed unpaired *t* test (*CtsZ*, *Dab2*, *Lyz*, *Reg3ɣ*, *Gip*, *Nts*, *Gcg*, *Pyy*, *Sst*, *Sct*, and *Cck*) or the Mann–Whitney test because data were not distributed normally when assessed by the D’Agostino and Pearson normality test (*CtsL*, *CtsA*, *Mcoln3*, and *Reg3β*). Error bars indicate means ± SD (*CtsZ*, *Dab2*, *Lyz*, *Reg3ɣ*, *Gip*, *Nts*, *Gcg*, *Pyy*, *Sst*, *Sct*, and *Cck*) or medians with interquartile range (*CtsL*, *CtsA*, *Mcoln3*, and *Reg3β*). Levels of significance are indicated: ∗*P* < .05, ∗∗*P* < .01. *n* = 8–12 pups per group.

In agreement with the results of global expression analyses (Figure 4*B*), relative expression assessed by qRT-PCR of the intracellular digestion markers *CtsL*, *CtsZ*, *CtsA*, *Dab2*, and *Mcoln3* was decreased (Figure 6*A*) in the distal SI of AB-treated pups compared with control pups. Analysis of whole distal SI by qRT-PCR also showed increased relative expression of the innate defense markers *Reg3*β and *Reg3*ɣ after AB treatment (Figure 6*B*), but not of *Lyz1*. However, immunohistochemical analysis of Lyz1 in distal SI showed a higher number of lysozyme-1–expressing epithelial cells per crypt in AB-treated pups compared with control pups (Figure 6*C*). The number of goblet cells as shown by the detection of mucins using Alcian blue and periodic acid-Schiff staining remained comparable between both groups (Figure 6*D*).

Among the top significantly up-regulated genes in proximal SI epithelial cells after AB treatment were genes expressed in EECs, reflecting increased (trans)epithelial sensing (Figure 4*F*). This observation also was confirmed in an independent experiment by qRT-PCR analysis of whole proximal SI tissue for the genes *Gip*, *Nts*, *Gcg*, *Pyy, Sst*, and *Sct*, except for *Cck* (Figure 6*E*). Overall, the genome-wide gene expression, qRT-PCR, and immunohistochemical data showed that early life AB treatment of mouse pups leads to a precocious maturation of the intestinal epithelium and induces expression of enteroendocrine markers in the proximal SI tissue.

### Mouse Intestinal Fetal Organoids Show the Direct Effects of Early Life Antibiotics on Intestinal Epithelial Maturation and Differentiation of Enteroendocrine Cells

**Figure 7:**
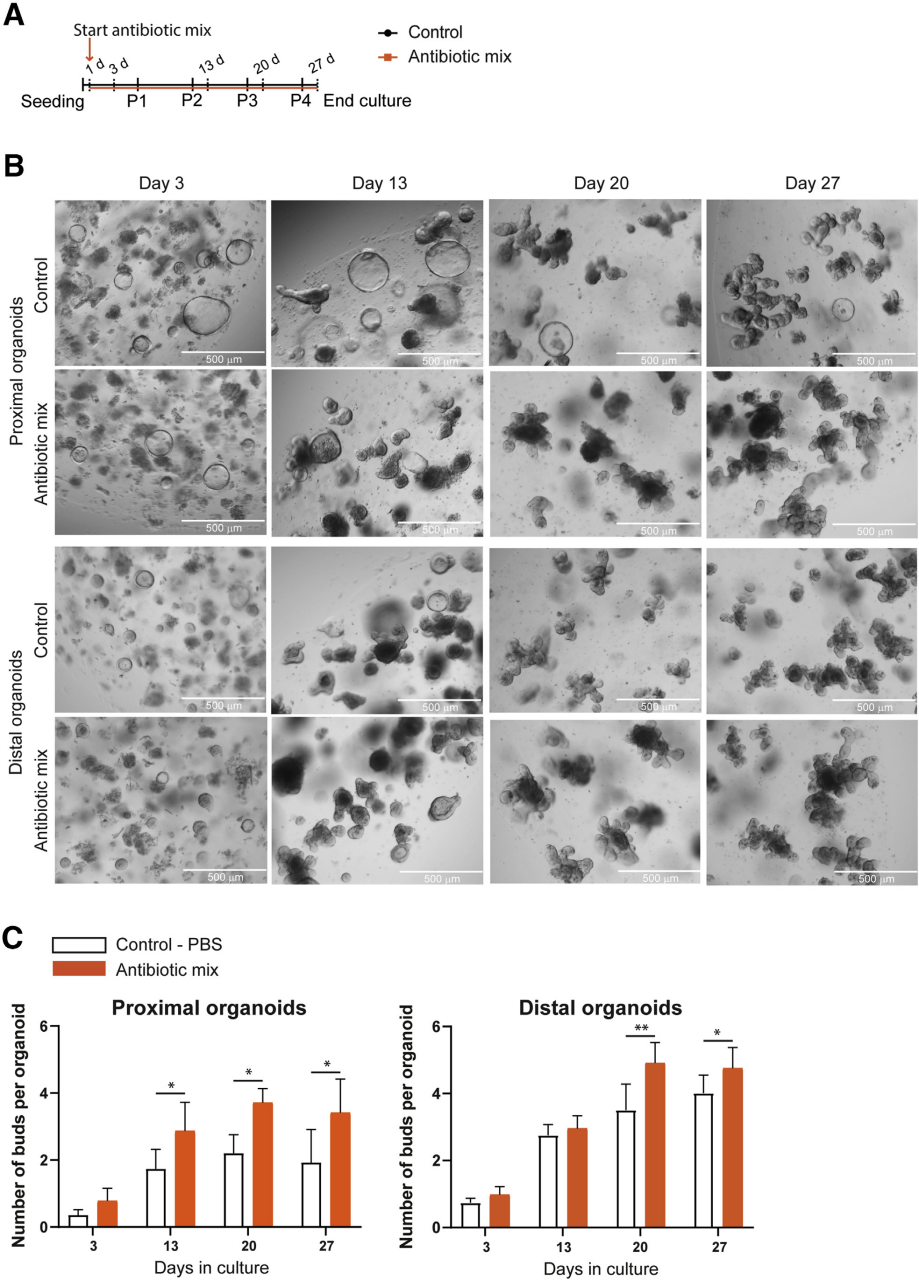
**Appearance and budding quantification of proximal and distal fetal intestinal organoids treated with early life antibiotics over time.** (*A*) Experimental design of in vitro treatment of mouse fetal intestinal organoids with the antibiotic mix. Organoids were analyzed on days 3, 13, 20, and 27 of culture. (*B*) Microscopic images of control and antibiotic mix–treated organoids on days 3, 13, 20, and 27 of culture. *Scale bars*: 500 μm. (*C*) Quantification of the number of buds per organoid of proximal and distal cultures in control and antibiotic mix conditions. Statistical analysis was performed by a 1-tailed paired *t* test. Error bars indicate means ± SD. Levels of significance are indicated: ∗*P* < .05, ∗∗*P* < .01). *n* = 18–57 organoids per condition and day of 4 independent cultures.

Intestinal epithelial maturation can be recapitulated in vitro (during a course of 1 month), using mouse fetal intestinal organoids.^80^ To study whether the changes in intestinal epithelial maturation we observed in vivo are a direct effect of the ABs on intestinal epithelial cells, we used fetal intestinal organoids from embryonic stage 19. We separately cultured the proximal and distal SI fetal organoids in the presence or absence of the same AB mix (amoxicillin, vancomycin, and metronidazole) as used in vivo (Figure 7*A*). Immature fetal organoid cultures present more spherical organoids,^89^^,^^90^ which are replaced over time by budding organoids as the cells mature in culture.^80^ The appearance of proximal and distal SI organoids was evidentially different, with less spherical organoids and more budding organoids observed in the culture treated with ABs compared with control (Figure 7*B*). Indeed, AB-treated organoids presented a higher number of budding structures compared with control organoids (Figure 7*C*).

**Figure 8:**
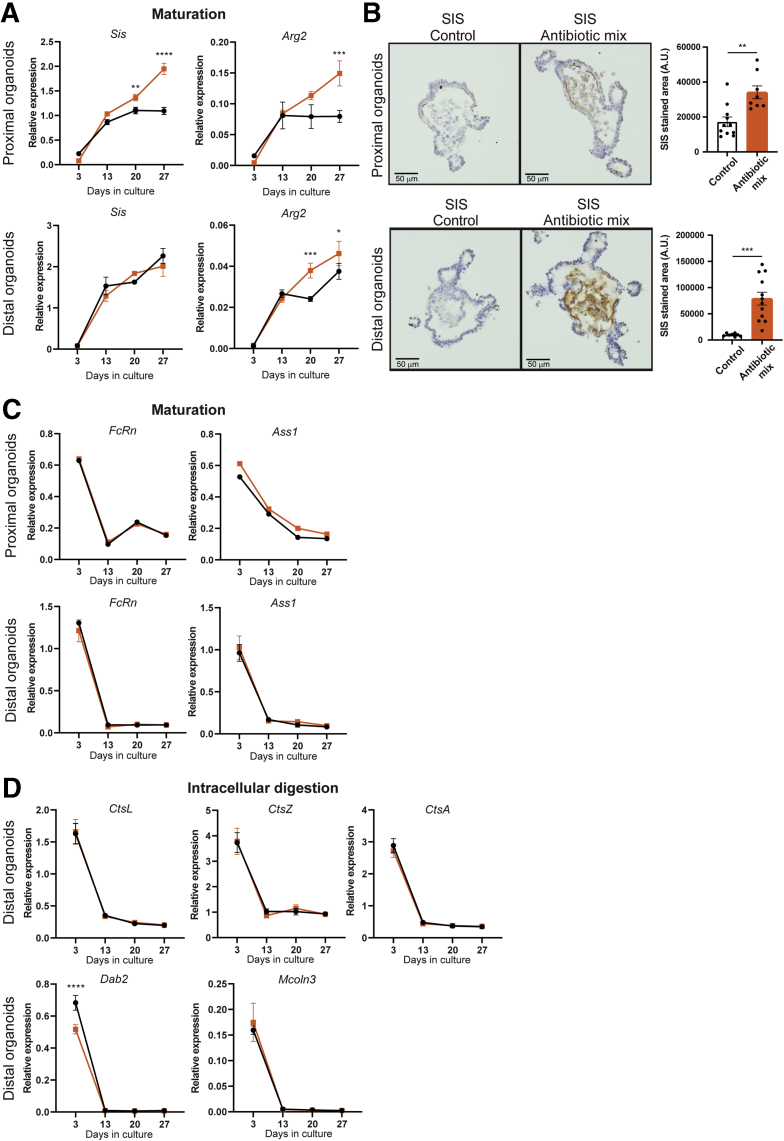
**Antibiotic treatment accelerates fetal organoid maturation in vitro.** (*A*) Relative expression of adult maturation markers *Sis* and *Arg2* detected by real-time qPCR in proximal and distal fetal organoids. Relative expression to reference genes *Rpl32* and *TbP*. (*B*) Immunohistochemistry and quantification of proximal and distal fetal organoids of SIS. *Scale bars*: 50 μm. Relative expression by real-time qPCR of (*C*) fetal maturation markers *FcRn* and *Ass1* in proximal and distal fetal organoids and of (*D*) intracellular digestion markers *CtsL*, *CtsZ*, *CtsA*, *Dab2*, and *Mcoln3* in distal fetal organoids. Relative expression to reference genes *Rpl32* and *TbP*. Statistical analysis was performed by 2-way analysis of variance with the (*A*, *C*, and *D*) Sidak multiple comparisons test or the (*B*) 2-tailed unpaired *t* test. Error bars indicate means ± SD. Levels of significance are indicated: ∗*P* < .05, ∗∗*P* < .01, ∗∗∗*P* < .001, ∗∗∗∗*P* < .0001. (*A*, *C*, and *D*) *n* = 3 individual wells from a representative organoid culture of 4–6 independent cultures and (*B*) *n* = 8–12 organoids of 2 independent cultures.

Comparable with our in vivo observations, gene expression analysis by qRT-PCR showed a significant up-regulation of adult markers *Sis* and *Arg2* by AB treatment in proximal SI fetal organoids, and of *Sis* also in distal SI fetal organoids, after 20 days of culture (Figure 8*A*). Accordingly, Sis protein levels were increased significantly by AB treatment in both proximal and distal SI organoids, as detected by immunohistochemistry (Figure 8*B*). The relative expression of neonatal markers *FcRn* and *Ass1* was not affected by the AB treatment in vitro (Figure 8*C*). There was no effect of the AB treatment in vitro on the gene expression level of *CtsL*, CtsZ, CtsA, *Dab2*, and *Mcoln3* in distal SI organoids, in contrast to what was shown in vivo (Figure 8*D*).

**Figure 9:**
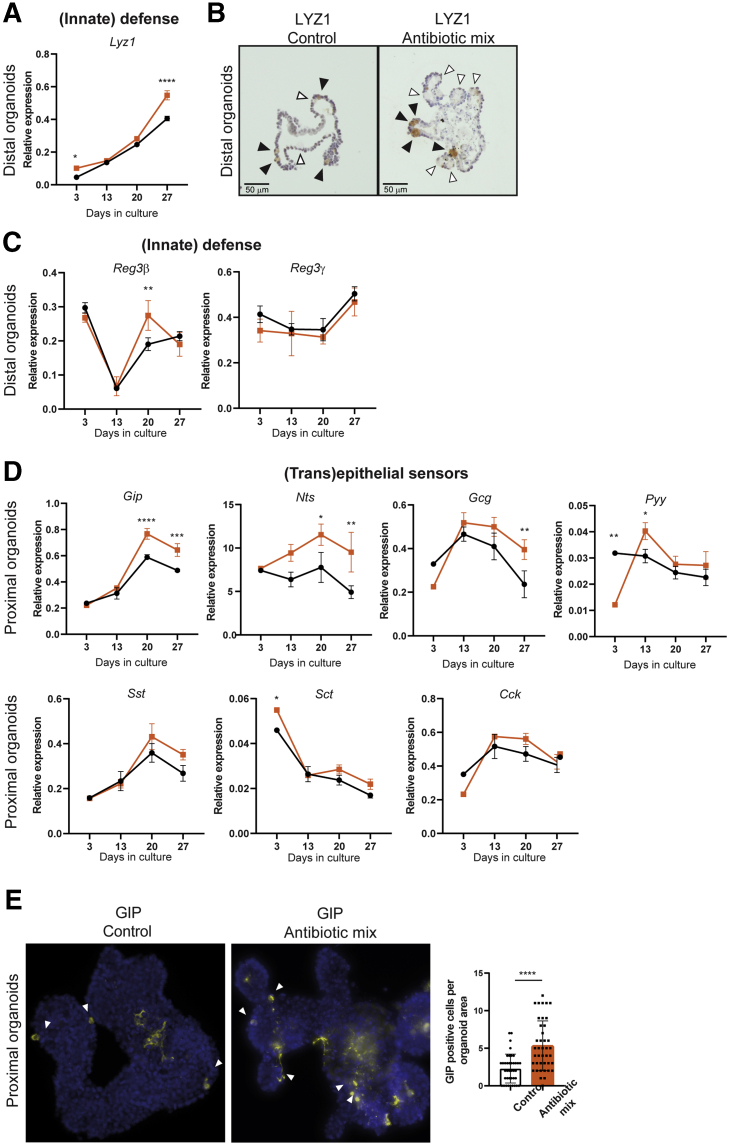
**Antibiotic treatment of in vitro fetal organoids induces differentiation of Paneth cells and enteroendocrine cells.** (*A*) Real-time qPCR analysis and (*B*) immunohistochemistry of innate defense marker *Lyz1*/LYZ1 in distal fetal organoids. Relative expression to reference genes *Rpl32* and *TbP*. *Black arrowheads* indicate Paneth cells positively stained for LYZ1 and *white arrows* indicate Paneth cells not stained for LYZ1. *Scale bars*: 50 μm. Real-time qPCR analysis of (*C*) innate defense markers *Reg3β* and *Reg3ɣ* in distal fetal organoids and (*D*) (trans)epithelial sensing markers *Gip*, *Nts*, *Gcg*, *Pyy*, *Sst*, *Sct*, and *Cck* in proximal fetal organoids. Relative expression to reference genes *Rpl32* and *TbP*. (*E*) Immunofluorescence of whole proximal fetal organoids for GIP and quantification of the amount of GIP-positive cells per organoid area. Statistical analysis was performed by (*A*, *C*, and *D*) 2-way analysis of variance with the Sidak multiple comparisons test or (*E*) 2-tailed unpaired *t* test. Error bars indicate means ± SD. Levels of significance are indicated: ∗*P* < .05, ∗∗*P* < .01, ∗∗∗*P* < .001, ∗∗∗∗*P* < .0001. (*A*, *C*, and *D*) *n* = 3 individual wells from a representative organoid culture of 4–6 independent cultures and (*G*) *n* = 40 organoids of 2 independent cultures.

The Paneth cell marker *Lyz1* was up-regulated in whole-genome expression analysis of distal IECs of the AB-treated group (Figure 4*E*). In vitro observations confirmed the significant increase in *Lyz1* expression after AB treatment in distal SI fetal organoids (Figure 9*A*), which was supported further by staining for the Lyz1 protein (Figure 9*B*). In contrast to Lyz1, the other markers of innate defense, *Reg3*β and *Reg3*ɣ, did not show the consistent increased gene expression levels in distal SI AB-treated organoids as with AB treatment in vivo (Figure 9*C*). Similar to our in vivo findings, proximal SI organoids treated with AB showed a significant increase in the expression of EEC markers *Gip*, *Nts*, *Gcg*, and *Pyy*, but not of the expression of *Sst*, *Sct*, and *Cck* (Figure 9*D*). To further examine this, expression of GIP was analyzed by immunostaining of proximal SI organoids, which showed a greater number of GIP-positive cells in proximal SI AB-treated organoids compared with control organoids (Figure 9*E*). In summary, these results indicate that AB can partially, but directly, affect the maturation of intestinal epithelial cells and differentiation of enteroendocrine cells.

### Antibiotics Directly Reduce the Metabolic Capacity of Fetal Intestinal Organoids

**Figure 10:**
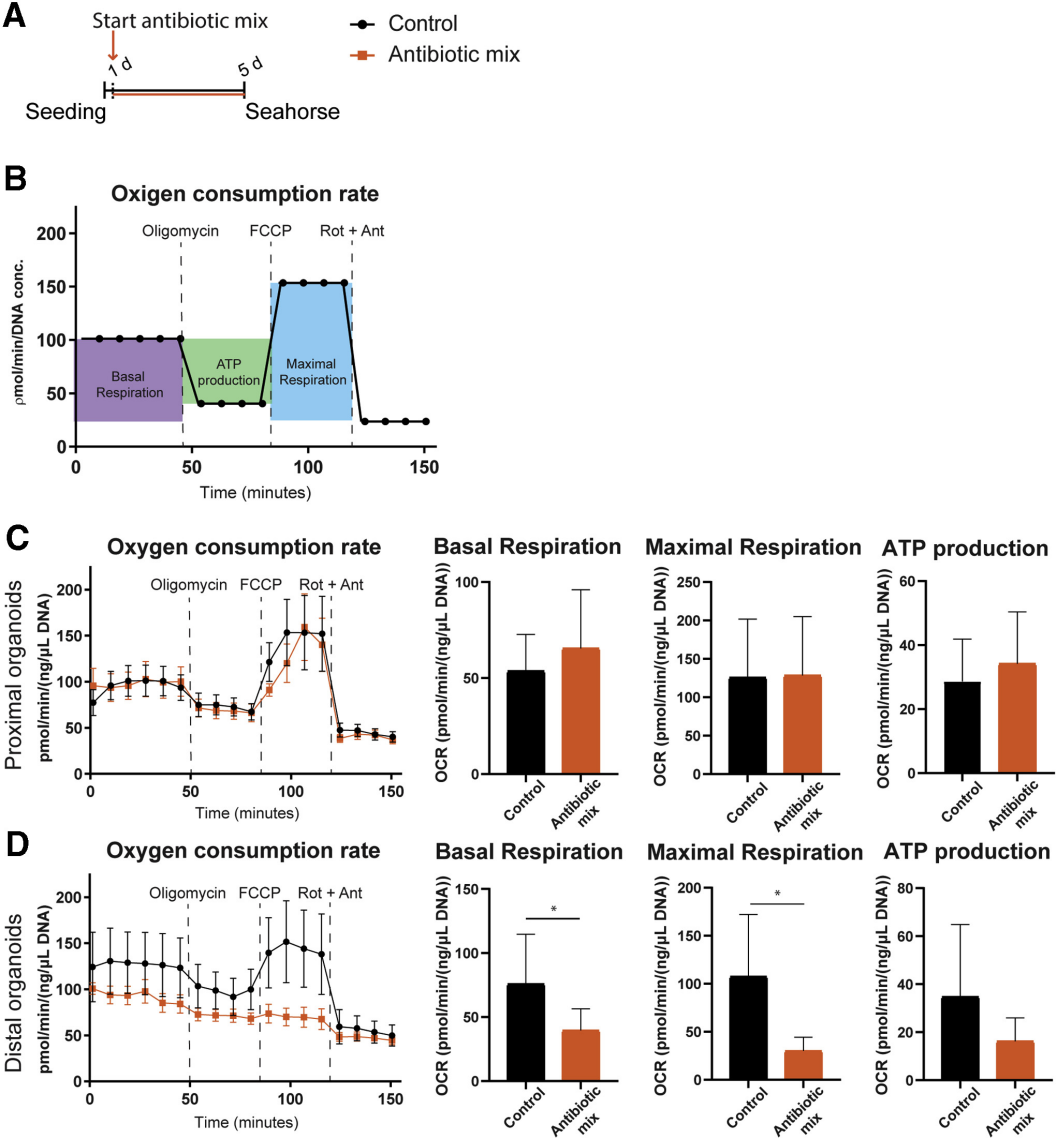
**Respiration capacity of distal fetal organoids decreases upon in vitro early life antibiotics.** (*A*) Experimental design of seahorse experiments to measure mitochondrial respiration and glycolysis in mouse fetal intestinal organoids after 5 days of antibiotic mix treatment. (*B*) Graphic representation of key parameters measured by OCR. Real-time respiration levels in the supernatant of (*C*) proximal and (*D*) distal fetal organoids measured as OCR, and basal respiration, ATP production, and maximal respiration rates calculated using OCR levels determined by seahorse assay. Statistical analysis was performed by the 1-tailed unpaired *t* test. Error bars indicate means ± SEM (*line graphs*) or means ± SD (*column graphs*). Levels of significance are indicated: ∗*P* < .05. *n* = 4–5 individual wells, representative of 2–4 independent experiments.

Several metabolic genes were changed in whole-genome expression analysis of IECs upon in vivo AB treatment (Supplementary Tables 1 and 2). In addition, different cells in the intestine have different metabolic profiles.^91^ To test whether ABs disturb the oxidative phosphorylation (or respiration) capacity of neonatal intestinal epithelial cells, we measured the real-time oxygen consumption of fetal organoids treated with the same mix of early life AB (amoxicillin, vancomycin, and metronidazole) for 96 hours (Figure 10*A*). Oligomycin, carbonyl cyanide-4 (trifluoromethoxy) phenylhydrazone (FCCP), and rotenone together with antimycin were added sequentially to the organoids to challenge the mitochondria and determine the amount of adenosine triphosphate (ATP) production and maximal capacity of mitochondrial respiration (Figure 10*B*). The oxygen consumption rate of proximal SI organoids was not affected by ABs (Figure 10*C*). Distal SI fetal organoids showed significantly lower basal respiration and impaired capacity for maximal respiration when treated with ABs, while ATP production followed the same trend (Figure 10*D*).

**Figure 11:**
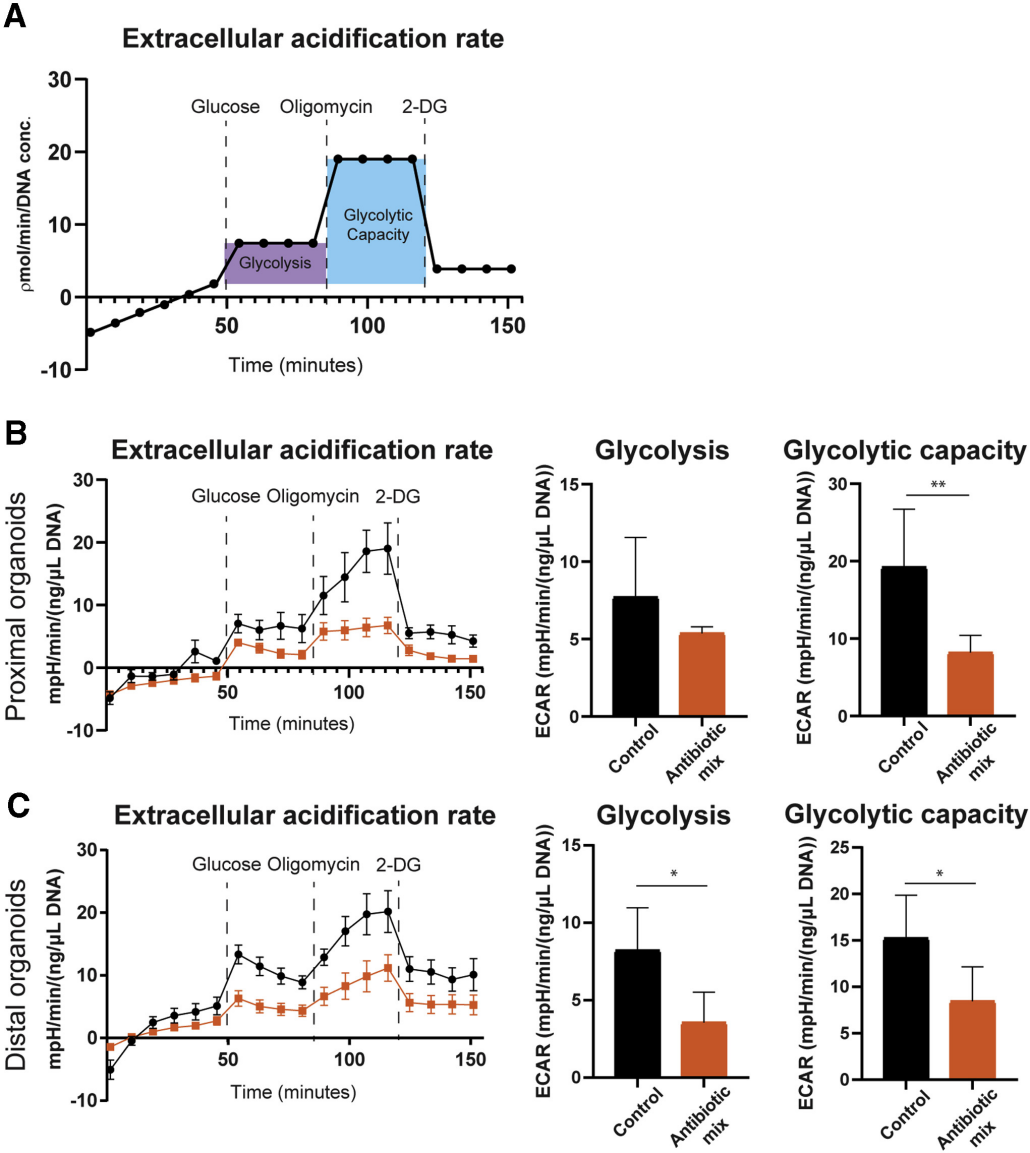
**Glycolytic capacity of fetal organoids decreases upon in vitro early life antibiotics.** (*A*) Graphic representation of key parameters measured by ECAR. Real-time glycolysis levels in the supernatant of (*B*) proximal and (*C*) distal fetal organoids measured as ECAR, and glycolysis and glycolytic capacity calculated using ECAR levels determined by seahorse assay. Statistical analysis was performed by the 1-tailed unpaired *t* test. Error bars indicate means ± SEM (*line graphs*) or means ± SD (*column graphs*). Levels of significance are indicated: ∗*P* < .05, ∗∗*P* < .01. *n* = 4–5 individual wells, representative of 2–4 independent experiments.

Next, we assessed real-time changes in extracellular acidification, a measurement of glycolysis in cells, to understand whether decreased respiration as a result of early life ABs is compensated for by an increase in glycolysis. Sequential addition of glucose, oligomycin, and 2-deoxy-glucose (2-DG) to the organoids allows the quantification of glycolysis rate and maximal glycolytic capacity (Figure 11*A*). Interestingly, we found that AB treatment significantly limited glycolysis in distal SI organoids and limited the glycolytic capacity in both proximal and distal SI organoids (Figure 11*B* and *C*). Together, our results show that AB treatment of fetal gut organoids directly affects the cellular metabolism of intestinal epithelial cells.

## Discussion

In this study, we showed that AB use during early life induces various changes in neonatal IECs. We found that AB exposure accelerates the maturation of the suckling intestinal epithelium, as shown by decreased gut permeability, the disappearance of vacuolated enterocytes, down-regulation of fetal/neonatal markers, and up-regulation of adult and (innate) defense markers (Figures 2 and 4). We observed decreased gut permeability with AB treatment in contrast to several previous studies that reported increased gut permeability after AB treatment.^44^^,^^55^^,^^92^^,^^93^ These studies were performed in adult mice, indicating that the developmental stage of the intestinal epithelium is an important factor determining the effect of the ABs. The reduced permeability we observed in the neonatal period could be owing to the loss of vacuolated enterocytes because the ACS contained in these cells can transport the FITC-dextran from the intestinal lumen to the basolateral side.^94^^,^^95^ The ACS is responsible for the intact transfer of milk immunoglobulins through the proximal intestinal epithelium.^68^^,^^69^ We could not find a difference in IgG and IgA serum levels upon AB treatment. The vacuolated cells are replaced by adult epithelium from the proximal SI enterocytes at approximately P15^65^^,^^69^ while they persist in the distal SI until weaning (P21),^96^ following/due to/because of a proximal to distal maturation wave.^63^ In our study, serum was analyzed at P20, which might represent a suboptimal time point to detect the differences in immunoglobulin uptake, because by then the vacuolated cells, responsible for immunoglobulin transfer, already have disappeared from the proximal SI. Nevertheless, our data show that the uptake of milk macromolecules by the intestinal epithelium of AB-treated mice is limited. Further studies with long-term follow-up evaluation are required to confirm whether precocious loss of vacuolated enterocytes has consequences in adult life or under disease conditions.

Global gene expression analyses showed a clear differential gene expression in the small intestine epithelium between control and AB-treated pups (Figures 3 and 4). The fact that greater differences were found in the distal SI compared with the proximal SI might be owing to the higher density of microbiota in the distal SI. ABs can strongly deplete the distal SI microbes, but some strains will survive the treatment, repopulate the intestinal lumen, and differently influence the intestinal epithelial cells. In addition, because of the proximal-to-distal wave of epithelial maturation that occurs during the suckling-to-weaning transition, proximal maturation is mostly completed by P20. Importantly, several differences in global gene expression were confirmed by qRT-PCR, and immunostaining, in an independent experiment (Figures 5 and 6). Collectively, these results indicate precocious maturation of the intestinal epithelium after treatment with early life ABs. Increased sensitivity to induced colitis in mice has been described after AB treatment.^37^^,^^45^^,^^48^^,^^52^^,^^92^ Recently, Al Nabhani et al^48^ showed that metronidazole and vancomycin depleted bacterial strains essential for the induction of the weaning reaction. Here, we provide evidence that AB treatment not only disturbs the development of the microbiota, and, consequently, the development of the immune system, but also affects the maturation process of the intestinal epithelial cells, which can contribute to the negative long-term effects. Moreover, our in vitro studies on fetal organoids showed that acceleration of epithelial maturation is partially a result of the direct action of ABs on the epithelial cells, indicated by the increased expression of adult maturation markers and Paneth cell marker lysozyme-1 (Figure 7, Figure 8, Figure 9). These findings confirm our previous study in which we showed that the intrinsic maturation process of the intestinal epithelium can be modulated by external factors such as early life ABs, as shown here.

In this study, we showed that the up-regulation of enteroendocrine cell markers after AB treatment in early life is a direct effect of ABs (Figures 6 and 9). Enteroendocrine cells are pivotal for (trans)epithelial sensing of specific nutrients, microbiota structures, and metabolites in the gut, and for the translation of these signals in the modulation of specific processes, such as the release of pancreatic, gastric, and hepatic enzymes; glucose homeostasis; food intake; and intestinal motility. Previous studies also have shown increased serum EEC-produced hormones glucagon-like peptide-1, glucagon, and gastric inhibitory polypeptide levels in adult mice treated with ABs^35^^,^^46^ and an up-regulated expression of *Gcg*, *Pyy*, and *Cck* in the cecum.^46^ Zarrinpar et al^46^ attributed these changes to an indirect effect of AB, through the microbiota, on the epithelial cells. However, in our fetal organoid model, the increase in expression of the enteroendocrine cell markers suggests that, in the neonatal epithelium, the effect of AB on the expression of specific EEC markers is at least partly a direct effect. The increased (trans)epithelial sensing and altered lipid metabolism detected in our global gene expression analysis (Supplementary Tables 1 and 2) could be involved in the increased risk of developing obesity and diabetes later in life after (prolonged) use of AB in early life.^31^, ^32^, ^33^, ^34^, ^35^ Treatment of our fetal organoid model with ABs also showed that part of their effect was indirect. We observed no change in intracellular digestion and innate defense (except for lysozyme), or for a couple of maturation and (trans)epithelial sensing markers, as identified by our in vivo approach. This indicates that many effects of early life ABs on intestinal epithelial cells are caused by alterations in the microbiome.

Finally, this study provides further evidence that ABs can directly disturb intestinal epithelial cell metabolism (Figures 10 and 11), supporting previous studies. Morgun et al^57^ showed that several intestinal genes affected by AB treatment in germ-free mice were similarly changed upon AB treatment of conventional mice. These genes, identified as a result of the direct effect of AB treatment, were expressed mainly in the epithelium and belonged to mitochondrial gene categories (electron transport chain, oxidation-reduction, ATP biosynthesis, and cellular and mitochondrial ribosomes).^57^ Moreover, the number of mitochondria in intestinal epithelial cells treated with ABs was reduced,^57^ which supports the concept that mitochondria, being structurally and functionally similar to bacteria, are a target of AB. The decreased (maximal) respiration measured in distal SI fetal organoids is in agreement with earlier reports showing diminished mitochondrial membrane potential and functions in cell lines^56^^,^^97^ upon AB treatment. Ampicillin, belonging to the same class and with the same target as amoxicillin, reduces mitochondrial basal respiration and maximal respiratory capacity, as well as overall metabolic activity in the intestinal epithelial cell line Caco-2.^56^ Vancomycin has been shown to depolarize the mitochondrial membrane, inhibit the mitochondrial complex I activity, and induce the production of mitochondrial reactive oxygen species in renal and kidney epithelial cells.^98^, ^99^, ^100^ In addition, we found a reduction in the glycolytic rate in distal SI organoids after AB treatment and reduced glycolytic capacity in AB-treated proximal and distal SI organoids. The decreased oxygen concentration along the proximal-to-distal small intestine^101^ and the changed expression of glucose transporters in the distal SI of AB-treated pups (Supplementary Table 2) might partly be responsible for the observed stronger effect of ABs on distal epithelial cells. The combination of reduced expression of *Slc2a2*/*Glut2*, which transports glucose at the basolateral membrane, down-regulation of *Slc37a4*, which is involved in the release of glucose to the blood upon glycogenolysis and gluconeogenesis, and up-regulation of *Slc2a1/Glut1*, responsible for basolateral uptake of glucose into the cell, seems to indicate that distal cells have limited access to glucose, leading to lower metabolic capacity. Therefore, the most probable mechanism for the observed decrease in intestinal metabolism upon AB treatment is the vancomycin-driven inhibition of mitochondrial complex I, together with the reduced capacity of glucose uptake. Furthermore, it previously was reported that adult intestinal organoids have a lower glycolytic capacity compared with Wnt-induced fetal organoids.^91^ The lower glycolytic rate induced by AB treatment of the fetal organoids might drive the precocious maturation we observed. More detailed studies are necessary to confirm this hypothesis and identify the possible mechanism.

NEC is one of the diseases associated with early life ABs.^23^, ^24^, ^25^ This severe disease, with long-term sequelae and mortality rates between 10% and 50%, is thought to affect preterm infants frequently because of their immature intestine.^102^ The accelerated and inappropriate maturation of the intestinal epithelium upon ABs, as well as the increased and inadequate (innate) intestinal defense, might explain the association between ABs and NEC in preterm infants. Further studies may elucidate which of the different types and combinations of ABs is responsible for the changes described in our study. Restricting the use to a single type or class, using different AB combinations, or adjusting the duration of treatment may reduce the risk of diseases, such as NEC, and prevent later disease development.

In conclusion, our study contributes to a better understanding of how AB administration early in life can indirectly and directly affect intestinal epithelial cells. Future investigations are needed to elucidate underlying mechanisms, to maximize the relevance and translational value of our findings.

## Methods

### In Vivo Studies

This study was conducted in accordance with institutional guidelines for the care and use of laboratory animals established by the Animal Ethics Committee of the University of Amsterdam*,* and all animal procedures related to the purpose of the research were approved under the Ethical license of the national competent authority, securing full compliance of the European Directive 2010/63/EU for the use of animals for scientific purposes.

Six pregnant 8-week-old C57Bl/6J females were obtained from Charles River and were allowed to adapt to the new environment for 1 week. Pregnant females were individually housed and received AIN-93G diet (Triple A Trading/Altromin, Tiel, The Netherlands). Mice were kept in Innovive disposable mice cages (San Diego, CA), enriched with corncob bedding and a carton house, with tissue as nesting material, under a strict 7 am to 7 pm dark-light cycle, controlled temperature and humidity, with food and water given ad libitum. Pups were monitored daily, weighted every other day from P10 onward, and kept with the mothers throughout the experiment. At P10, 2 experimental groups were defined randomly: the treatment group (3 litters, 4 pups per litter) received daily oral gavage of 30 μL ABs (25 mg/kg/day amoxicillin [Amsterdam University Medical Center Pharmacy], 50 mg/kg/day metronidazole [Amsterdam University Medical Center Pharmacy], and 50 mg/kg/day vancomycin [Sigma/Aldrich, St. Louis, MO]); the control group (3 litters, 4 pups per litter) received daily oral gavage of 30 μL phosphate-buffered saline (PBS). ABs or PBS were given consistently during the light period, always at the same time period of the day, nonblinded. For oral gavage, pups were separated from the mother all at once and placed back all at once as well, to correct for differences in maternal care. On P20, mothers were killed by CO_2_ and isoflurane exposure and pups were fasted. After 3 hours, 50 μL of 60 mg/100 g weight FITC-dextran 4 kilodaltons (Sigma/Aldrich) diluted in PBS were given via oral gavage to all the pups to assess intestinal permeability. After 4 hours, pups were euthanized by CO_2_ and isoflurane exposure. Immediately afterward, blood was collected by heart puncture in MiniCollect Z Serum Sep Clot tubes (Greiner, Kremsmünster, Austria). After 30 minutes of incubation on ice, in the dark, blood was centrifuged and serum was collected and kept at -80°C.

### FITC-Dextran In Vivo Permeability Assay

Standard samples were obtained by 2-fold serial dilution of 1 mg/mL FITC-dextran in blood serum. The fluorescence signals of the serum samples were recorded with an excitation wavelength of 485 nm and an emission wavelength of 520 nm and compared with the standard curve values. The amount of FITC-dextran in serum samples was calculated in nanograms per milliliter.

### In Vitro Studies

Per the experiment, 2 pregnant 8-week-old C57Bl/6J mice were obtained from Charles River (Sulzfeld, Germany), housed together, and were killed by CO_2_ and isoflurane exposure on day 19 of the pregnancy. To generate fetal organoid culture, fetuses from 2 mice were combined, resulting in a final number of approximately 12–15 fetuses per experiment. Fetal small intestine tissue was harvested, separated into proximal and distal parts, dissociated, and cultured as previously described in a 48-well plate.^80^^,^^81^ Fetal organoids were maintained in Epidermal Growth Factor, Noggin, Rspo1 (ENR) medium, without penicillin and streptomycin, throughout the experiments. On day 1 of culture, 15 μg/mL amoxicillin (Amsterdam University Medical Center pharmacy), 25 μg/mL metronidazole (Amsterdam University Medical Center pharmacy), and 50 μg/mL vancomycin (Sigma/Aldrich) were added to half of the wells. The medium was refreshed 3 times per week. Samples for RNA analyses or immunostaining were always taken 3 days after passaging of the culture. Representative images of the cultures were taken by an inverted light microscope (Leica, Wetzler, Germany).

### Immunostaining

Tissue was flushed with PBS, fixed overnight in 4% formaldehyde, embedded in paraffin, and sectioned. Sections were deparaffinized with xylene and gradually rehydrated in ethanol. After blocking the endogenous peroxidase (0.01% H_2_O_2_ in methanol; only performed for immunohistochemistry), slides were boiled in 0.01 mol/L sodium citrate buffer (pH 6) for 10 minutes at 120ºC in an autoclave for antigen retrieval. Slides were blocked for 30 minutes at room temperature in PBS with 1% bovine serum albumin and 0.1% Triton X-100 (Sigma, St. Louis, MO). Then, slides were incubated overnight with primary antibody diluted in the blocking buffer. Slides were washed with PBS and secondary antibody diluted in blocking buffer was added for 30 minutes at room temperature. For immunofluorescence, slides were mounted using Prolong Gold antifade reagent with 4′,6-diamidino-2-phenylindole (Invitrogen, Waltham, MA). For immunostaining, antibody binding was visualized by adding chromagene substrate diaminobenzidine (Sigma-Aldrich), sections were counterstained using hematoxylin (Sigma), and slides were dehydrated and mounted with Entellan (VwR, Leicestershire, UK).

Immunohistochemistry in fetal organoids was performed as previously described.^81^^,^^103^ For whole-mount immunofluorescence staining, organoids were collected from the Matrigel by Cell Recovery Solution (Corning B.V., Amsterdam, The Netherlands) and fixed for 1 hour in 4% formaldehyde. After washing (PBS + glycine), permeabilization (PBS + 0.5% Triton X-100), and blocking (Immunofluorescen-wash + 10% goat serum), organoids were incubated with primary antibody diluted in blocking buffer for 1–2 hours at room temperature. Staining was visualized with Alexa-conjugated secondary antibody (1 hour at room temperature), after which cells were mounted on a slide with ProLong Gold antifade with 4′,6-diamidino-2-phenylindole reagent (Invitrogen). Sections were examined using a brightfield microscope (BX51; Olympus, Leiderdorp, The Netherlands) or a fluorescence microscope (DM-6000B; Leica).

The following antibodies were used for immunostaining: Ki67: rabbit polyclonal anti-Ki67 (1:4000, ab15580; Abcam, Cambridge, UK); phospho-histone H3: rabbit polyclonal anti-phospho-histone H3 (Ser10) (1:200, PAS-17869; Thermo Fisher Scientific, Waltham, MA); SIS: rabbit monoclonal antisucrase isomaltase (C-8) (1:200, sc-393470; Santa Cruz, Heidelberg, Germany); ASS1: rabbit polyclonal anti-argininosuccinate synthase I (1:15,000)^104^; LYZ1: rabbit polyclonal anti-lysozyme (1:2000, A0099; DAKO, Santa Clara, CA); GIP: rabbit polyclonal antigastric inhibitory peptide (1:500, ab22624; Abcam); and AF488: goat polyclonal anti-rabbit (1:500, A11008; Invitrogen).

### Immunoglobulin Detection

Immunoglobulin detection was performed in serum samples using IgG and IgA total Enzyme-Linked Immunosorbent Assay Ready-SET-Go! kit (Affymetrix eBioscience, Waltham, MA) and according to the manufacturer’s protocol.

### Epithelial Cell Fluorescence-Activated Cell Sorting

The small intestine of P20 pups was cut open and proximal and distal parts were separated, cut into pieces, and washed with ice-cold PBS. Intestines of 4 different pups, belonging to the same litter, were combined into 1 sample used for transcriptome profile analysis. Crypts were dissociated after incubation with 2 mmol/L EDTA (Merck/VWR) for 30 minutes at 4°C and filtered through a 70-μm cell strainer (BD/VWR). Single cells were obtained by incubating crypts with TrypLE Express (Invitrogen). Cells were kept in PBS 2% fetal calf serum rho-kinase inhibitor and RNase inhibitor (Fermentas, Vilnius, Lithuania/Thermo Fisher Scientific) solution and stained with EpCAM-FITC antibody (1:50, 324204; BioLegend, San Diego, CA) for 30 minutes on ice.

### RNA Isolation and qRT-PCR

For transcriptome profiling, RNA was extracted from EpCAM-positive cells using the phenol–chloroform method. RNA quality was measured on an Agilent 2100 Bioanalyzer (Palo Alto, CA).

For qRT-PCR, RNA from whole-tissue tissue and fetal organoids was isolated using the Bioline ISOLATE II RNA Mini kit (BIO-52073; Bioline, Cincinnati, OH) according to the manufacturer’s instructions. A total of 1 μg (tissue) or 0.3 μg (organoids) RNA was transcribed using Revertaid reverse transcriptase according to the protocol (Fermentas, Vilnius, Lithuania). qRT-PCR was performed on a BioRad iCycler (Hercules, CA) using sensifast SYBR No-ROX Kit (Bio-98020; GC biotech, Waddinxveen, The Netherlands) according to the manufacturer’s protocol. The 2 most stable reference genes were determined using GeNorm, and their geometric mean was used to calculate the relative expression of genes of interest: for whole-tissue qRT-PCR cyclophilin (*Cyp*) and peptidylprolyl isomerase B (*Ppib*) and for fetal organoids qRT-PCR ribosomal protein L32 (*Rpl32*) and TATA-box binding protein (*TbP*). Relative gene expression was calculated using N0 values obtained by LinRegPCR analysis. Primers were validated previously using melting curve analyses and gel electrophoresis of PCR products. The following primers were used: *Cyp*: forward: ATGGTCAACCCCACCGTGT; reverse: TTCTGCTGTCTTTGGAACTTTGTC; *Ppib*: forward: GCCAACGATAAGAAGAAGGGA; reverse: TCCAAAGAGTCCAAAGACGAC; *Tbp*: forward: GGGTATCTGCTGGCGGTTT; reverse: TGGAAGGCTGTTGTTCTGGT; *Rpl32*: forward: TGGAGGTGCTGCTGATGTG; reverse: GCGTTGGGATTGGTGACTCT; *Sis*: forward: TGCCTGCTGTGGAAGAAGTAA; reverse: CAGCCACGCTCTTCACATTT; *Arg2*: forward: TAGGGTAATCCCCTCCCTGC; reverse: AGCAAGCCAGCTTCTCGAAT; *FcRn*: forward: CTTCAGGCGCATAGACGG; reverse: CTAAACTCTTGTCCGGAGCG; *Ass1*: forward: CATTGGAATGAAGTCCCGAG; reverse: GATTTTGCGTACTTCCCGAT; *CtsL*: forward: CGACTGTGGGGCCTATTTCT; reverse: ATAGCCCACCAACAGAACCC; *CtsZ*: forward: CTACCAGGCCAAGGACCAAG; reverse: GCCATTATCCCGCAGCTGAT; *CtsA*: forward: GCTAGTGGACTACGGGGAGA; reverse: GTGTCCGGCACCCTTGATG; *Dab2*: forward: TCATCAAACCCCTCTGTGGT; reverse: AGCGAGGACAGAGGTCAACA; *Mcoln3*: forward: TTTTGCGGATGGATTGTGCT; reverse: TATCAGCGAGAACAGGCACTC; *Lyz*: forward: GGATGGCTACCGTGGTGTCAAGC; reverse: TCCCATAGTCGGTGCTTCGGTC; *Reg3β*: forward: TGGGAATGGAGTAACAAT; reverse: GGCAACTTCACCTCACAT; *Reg3ɣ*: forward: CCATCTTCACGTAGCAGC; reverse: CAAGATGTCCTGAGGGC; *Gip*: forward: AACTGTTGGCTAGGGGACAC; reverse: TGATGAAAGTCCCCTCTGCG; *Nts*: forward: TGCTGACCATCTTCCAGCTC; reverse: GAATGTAGGGCCTTCTGGGT; *Gcg*: forward: CTTCCCAGAAGAAGTCGCCA; reverse: GTGACTGGCACGAGATGTTG; *Pyy*: forward: ACGGTCGCAATGCTGCTAAT; reverse: GCTGCGGGGACATCTCTTTTT; *Sst*: forward: GACCTGCGACTAGACTGACC; reverse: CCAGTTCCTGTTTCCCGGTG; *Sct*: forward: GACCCCAAGACACTCAGACG; reverse: TTTTCTGTGTCCTGCTCGCT; and *Cck*: forward: GAAGAGCGGCGTATGTCTGT; reverse: CCAGAAGGAGCTTTGCGGA.

### Transcriptome Profiling

For transcriptome profiling, 400 ng RNA was amplified and labeled using the 3’ IVT pico kit Affymetrix RNA amplification kit (Nugen, Redwood City, CA) according to the manufacturer’s protocol. Microarray analysis of mouse EpCAM-positive cells was performed using the Affymetrix Clariom S 8-Array HT Plate according to the standard protocols of the Dutch Genomics Service and Support Provider (MAD, Science Park, University of Amsterdam, The Netherlands). The data were uploaded and normalized using R2: Genomics Analysis and Visualization Platform (http://hgserver1.amc.nl) (Amsterdam, The Netherlands). Microarray results were analyzed using R2 software. Differentially expressed genes were selected based on fold change (≥2) in comparison with the control group. For gene set enrichment analysis, sets of genes from the Molecular Signatures database (http://software.broadinstitute.org/gsea/msigdb/genesets.jsp) and the GEO GSE92332^88^ data set were extracted and signatures of HALLMARK Vacuole organization, GO Fatty acid metabolism, proximal and distal enterocytes, and proximal and distal enteroendocrine cells were compared with our data set using GSEA software (http://software.broadinstitute.org/gsea/index.jsp),

### Seahorse Analysis of Fetal Organoids

Fetal organoids were treated with ABs described earlier, starting from day 1 of culture and transferred to a XFe24 cell culture microplate (Agilent, Palo Alto, CA) on day 3 of culture, with new medium containing the same antibiotics. After 2 days, organoid wells were washed twice with PBS, assay medium (Agilent) was added, and organoids were kept without CO_2_ until the start of the measurements. The oxygen consumption rate and extracellular acidification rate were measured in an XF24 seahorse machine (Agilent) according to the manufacturer’s instructions. Immediately afterward, organoids were collected from the Matrigel (Corning B.V., Amsterdam, The Netherlands) using Cell Recovery Solution (Corning B.V., Amsterdam, The Netherlands), and DNA was extracted and measured in nanodrop (Thermo Fisher Scientific) to normalize experimental values.

The assay medium used was Dulbecco’s modified Eagle medium (D5030; Sigma-Aldrich), with 4 mmol/L glutamine and, for the oxygen consumption rate (OCR) assay, with 10 mmol/L glucose.

The concentration of injected compounds was 1 μmol/L oligomycin, 0.5 μmol/L FCCP, 1 μmol/L rotenone, 1 μmol/L antimycin A, 10 mmol/L glucose, and 50 mmol/L 2-DG.

The compounds used to challenge mitochondrial respiration were as follows: oligomycin represses ATP production in the mitochondria, decreasing OCR; FCCP stimulates respiration to the maximum capacity of the mitochondria, increasing OCR; rotenone and antimycin A completely shuts down respiration, decreasing OCR.

The compounds used to challenge glycolysis were as follows: glucose is added to the glucose-depleted medium, increasing the extracellular acidification rate (ECAR) to basal conditions, oligomycin shifts ATP production from mitochondrial respiration to glycolysis, increasing ECAR, and 2-DG inhibits glycolysis, decreasing ECAR.

Basal respiration was calculated by subtracting the minimum rate after adding rotenone and antimycin A from the last rate measurement before adding oligomycin. ATP production was calculated by subtracting the minimum rate measurement after adding oligomycin from the last rate measurement before adding oligomycin. Maximal respiration was calculated by subtracting the minimum rate after adding rotenone and antimycin A from the maximum rate measurement after adding FCCP.

Glycolysis was calculated by subtracting the last rate measurement before adding glucose from the maximum rate measurement before adding oligomycin. Glycolytic capacity was calculated by subtracting the last rate measurement before adding glucose from the maximum rate measurement after adding oligomycin.

### Software

The software used was as follows: nQuery 7.0 (San Diego, CA) for sample-size calculations; ImageJ (National Institutes of Health, Bethesda, MD) for villi length and crypt depth measurement, SIS immunostaining quantification; Transcriptome Analysis Console (Thermo Fisher Scientific) for microarray data analysis, principal component analysis, and volcano plots creation and identification of 2-fold differentially expressed genes; R2 for heatmap creation; GSEA software (http://software.broadinstitute.org/gsea/index.jsp) for GSEA analysis; LinRegPCR for quantitative real-time PCR analysis; GeNorm for identification of most stable reference genes for quantitative real-time PCR analysis; and GraphPad Prism 8 (San Diego, CA) for statistical analysis and graph creation.

### Statistical Analysis

Sample size was calculated using nQuery and based on maturation studies, using a 2-group *t* test of equal n, with a significance level (α) of 0.05 and power of 80%. There were no exclusions or drop-outs to report. Analysis was performed with blinded data. Data were analyzed using GraphPad Prism 8 and are presented as means ± SD unless stated otherwise in the figure legends. Sample distribution was determined using the D’Agostino and Pearson normality test. Sample numbers, experimental replicates, type of statistical analysis test, and *P* values are reported in the figure legends. Two-way analysis of variance was used for time-course experiments of nonrepetitive measurements because differences at each time point were analyzed.

### Data Availability

Microarray data have been deposited in the Gene Expression Omnibus Database (GSE172061).

All authors had access to the study data and reviewed and approved the final manuscript.

## CRediT Authorship Contributions

Tânia Martins Garcia (Conceptualization: Lead; Data curation: Lead; Formal analysis: Lead; Investigation: Lead; Methodology: Equal; Project administration: Lead; Resources: Supporting; Software: Equal; Visualization: Lead; Writing – original draft: Lead)

Manon van Roest (Investigation: Supporting; Methodology: Supporting; Validation: Equal; Writing – review & editing: Supporting)

Jacqueline Vermeulen (Investigation: Supporting; Methodology: Supporting; Validation: Equal; Writing – review & editing: Supporting)

Sander Meisner (Formal analysis: Supporting; Investigation: Supporting; Methodology: Supporting; Software: Supporting; Validation: Supporting; Writing – review & editing: Supporting)

Wouter L. Smit (Formal analysis: Supporting; Methodology: Supporting)

Joana Silva (Formal analysis: Supporting; Methodology: Supporting; Resources: Supporting; Writing – review & editing: Equal)

Pim J. Koelink (Investigation: Supporting; Methodology: Supporting; Resources: Supporting; Writing – review & editing: Equal)

Jan Koster (Resources: Supporting; Software: Supporting; Writing – review & editing: Equal)

William J. Faller (Methodology: Supporting; Resources: Equal; Writing – review & editing: Supporting)

Manon E. Wildenberg (Investigation: Supporting; Methodology: Supporting; Resources: Supporting; Supervision: Supporting; Writing – review & editing: Supporting)

Ruurd van Elburg (Funding acquisition: Equal; Methodology: Equal; Supervision: Equal; Writing – original draft: Equal)

Vanesa Muncan (Conceptualization: Lead; Data curation: Equal; Formal analysis: Equal; Funding acquisition: Equal; Methodology: Lead; Resources: Equal; Software: Equal; Supervision: Lead; Visualization: Lead; Writing – original draft: Equal)

Ingrid B. Renes (Conceptualization: Lead; Funding acquisition: Equal; Methodology: Equal; Resources: Supporting; Supervision: Lead; Visualization: Supporting; Writing – original draft: Equal)

The authors want to thank the staff from ARIA and the research team DigeST Amsterdam for their help.

## Figures and Tables

**Supplementary Table 1:** Up-regulated and Down-regulated Genes in Whole-Genome Analysis of Proximal Epithelial Cells Treated With In Vivo AB Treatment, Compared With Control

Gene symbol	*P* value	Fold change	Transcript cluster ID
*Nts*	.0288	12.36	TC1000002780.mm.2
*Sst*	.0104	10.66	TC1600001398.mm.2
*Afp*	.009	5.94	TC0500000835.mm.2
*Scg2*	.0174	5.21	TC0100002640.mm.2
*Sct*	.0066	4.68	TC0700004532.mm.2
*Iapp*	.0036	4.6	TC0600001677.mm.2
*Rbp4*	.0124	4.59	TC1900001464.mm.2
*Cpe*	.0074	4.48	TC0800002310.mm.2
*Ambp*	.023	3.73	TC0400002796.mm.2
*Gip*	.0185	3.67	TC1100001480.mm.2
*Cck*	.0494	3.67	TC0900003266.mm.2
*Alox5*	.0087	3.54	TC0600002975.mm.2
*Cyp2b10*	.0101	3.51	TC0700000430.mm.2
*Gcg*	.0108	3.5	TC0200003628.mm.2
*Trpm5*	.0455	3.31	TC0700004579.mm.2
*Sh2d6*	.0081	3.24	TC0600002493.mm.2
*Serpina1c*	.0178	3.24	TC1200002534.mm.2
*Pyy*	.0134	3.22	TC1100003823.mm.2
*Sez6l2*	.0232	3.09	TC0700004626.mm.2
*Resp18*	.0335	3.08	TC0100002607.mm.2
*Cybrd1*	.0485	3.08	TC0200001032.mm.2
*Cacna1a*	.039	3.05	TC0800000955.mm.2
*Vav1*	.0298	2.91	TC1700001033.mm.2
*Gm21887*	.0127	2.88	TC0X00001715.mm.2
*Chga*	.0332	2.88	TC1200001018.mm.2
*Gm21845*	.0496	2.85	TC0Y00000069.mm.2
*Gm21350;* *LOC100861950*	.0496	2.85	TC0Y00000071.mm.2
*Gm21791*	.0496	2.85	TC0Y00000073.mm.2
*Gm21890*	.0496	2.85	TC0Y00000075.mm.2
*Dclk1*	.0377	2.8	TC0300000419.mm.2
*Reg3a*	.0045	2.72	TC0600000876.mm.2
*AA467197;* *Mir147*	.0316	2.71	TC0200001854.mm.2
*Kctd12;* *Mir5130*	.049	2.67	TC1400002632.mm.2
*Ptpn6*	.0487	2.66	TC0600003114.mm.2
*Hbb-bs; Hbb-b1*	.0236	2.65	TC0700003909.mm.2
*Ptgs1*	.0243	2.57	TC0200000651.mm.2
*Slc38a11*	.0394	2.55	TC0200003662.mm.2
*Pip4k2a*	.0089	2.54	TC0200003051.mm.2
*Erdr1*	.0091	2.35	TC0Y00000223.mm.2
*Scnn1a*	.0292	2.34	TC0600001458.mm.2
*Stxbp5l*	.0238	2.33	TC1600001590.mm.2
*Fap*	.0059	2.29	TC0200003629.mm.2
*Scg3*	.0056	2.26	TC0900002681.mm.2
*Maoa*	.0268	2.25	TC0X00000181.mm.2
*Slc14a1*	.0156	2.23	TC1800001637.mm.2
*Upp1*	.02	2.2	TC1100000119.mm.2
*Hbb-bt; Hbb-b2*	.0056	2.19	TC0700003908.mm.2
*Pik3cg*	.0067	2.14	TC1200001605.mm.2
*BC089597*	.0076	2.12	TC1000001576.mm.2
*Sh2d7*	.0139	2.07	TC0900000658.mm.2
*Olfr814*	.0242	2.07	TC1000003194.mm.2
*Sval1*	.0035	2.05	TC0600000448.mm.2
*Ifit1bl1*	.048	2.04	TC1900001422.mm.2
*Fam110b*	.0028	-2.09	TC0400000032.mm.2
*Hao2*	.0071	-2.19	TC0300002566.mm.2
*Renbp*	.0431	-2.23	TC0X00002440.mm.2
*Sparc*	.0151	-2.5	TC1100002783.mm.2
*Smcp*	.0202	-2.79	TC0300002397.mm.2
*Paqr9*	.0345	-2.84	TC0900001195.mm.2
*Hif3a*	.0103	-2.91	TC0700002445.mm.2
*Spp2*	.0459	-2.91	TC0100000847.mm.2
*Gpx3*	.0358	-3.04	TC1100000638.mm.2
*Folr1*	.0111	-3.45	TC0700003846.mm.2
*Hyal5*	.0222	-4.16	TC0600000206.mm.2
*Dab2*	.0075	-4.3	TC1500000031.mm.2
*Npl*	.0111	-5.34	TC0100003280.mm.2
*Lgmn*	.0026	-5.35	TC1200002251.mm.2

**Supplementary Table 2:** Up-regulated and Down-regulated Genes in Whole-Genome Analysis of Distal Epithelial Cells Treated With In Vivo AB Treatment, Compared With Control

Gene symbol	*P* value	Fold change	Transcript cluster ID
*Spp2*	1.51E-07	-32.87	TC0100000847.mm.2
*Ctrb1*	1.18E-07	-22.25	TC0800002951.mm.2
*Rab30*	.0014	-15.02	TC0700001317.mm.2
*Axl*	4.16E-07	-14.31	TC0700002644.mm.2
*Hyal5*	6.00E-05	-13.36	TC0600000206.mm.2
*C2*	9.63E-07	-13.26	TC1700002817.mm.2
*Slc43a3*	1.14E-05	-12.63	TC0200001226.mm.2
*Ctsl*	2.44E-05	-12.49	TC1300002204.mm.2
*St3gal5*	1.41E-05	-12.22	TC0600000841.mm.2
*Aph1c*	3.58E-06	-11.24	TC0900002532.mm.2
*Csf1r*	2.80E-05	-10.8	TC1800000625.mm.2
*Bglap3*	2.90E-05	-10.3	TC0300003235.mm.2
*Ass1; Gm5424*	.0002	-10.21	TC0200000551.mm.2
*Slc46a3*	8.25E-05	-10.09	TC0500003632.mm.2
*Gpx3*	.0008	-10.09	TC1100000638.mm.2
*Galns*	6.16E-06	-9.65	TC0800003077.mm.2
*Fcgrt*	.0001	-9.22	TC0700002989.mm.2
*Gml*	.001	-9.05	TC1500001721.mm.2
*Trim30a*	.0016	-8.38	TC0700003943.mm.2
*Tmem9*	1.28E-06	-7.59	TC0100001258.mm.2
*Dab2*	5.62E-05	-7.54	TC1500000031.mm.2
*Neu1*	6.05E-06	-7.48	TC1700000652.mm.2
*Lgmn*	5.67E-05	-7.37	TC1200002251.mm.2
*Lipa*	.0011	-7.31	TC1900001419.mm.2
*Apoc2; Apoc4*	.0047	-7.26	TC0700002495.mm.2
*Man2b2*	8.40E-06	-7.23	TC0500002266.mm.2
*Bglap2*	.0001	-7.15	TC0300003236.mm.2
*Slc40a1*	6.37E-05	-7.12	TC0100002285.mm.2
*Aqp7*	.0062	-7.11	TC0400002455.mm.2
*Bglap*	3.51E-06	-6.65	TC0300002309.mm.2
*Mcoln3*	.0007	-6.57	TC0300001488.mm.2
*Pid1*	6.13E-05	-6.56	TC0100002690.mm.2
*Slc19a3*	2.18E-05	-6.52	TC0100002681.mm.2
*Renbp*	.0001	-6.35	TC0X00002440.mm.2
*Igf2*	4.01E-05	-6.3	TC0700004571.mm.2
*Npl*	.0011	-6.18	TC0100003280.mm.2
*Rhod*	.0003	-6.17	TC1900000921.mm.2
*Rhoc*	.0011	-6.16	TC0300001081.mm.2
*Clca4c-ps*	.0055	-6.1	TC0300003081.mm.2
*Wwc2*	6.26E-05	-5.94	TC0800002184.mm.2
*Gabrb3*	1.38E-05	-5.92	TC0700000941.mm.2
*Acot12*	.0043	-5.9	TC1300001028.mm.2
*Mfi2*	.0002	-5.87	TC1600000421.mm.2
*Irf7*	.0002	-5.83	TC0700004530.mm.2
*Tmem140*	.0081	-5.81	TC0600000340.mm.2
*Rtp4*	.0004	-5.77	TC1600000346.mm.2
*Gm10768; Abcc2*	5.58E-05	-5.73	TC1900000637.mm.2
*Pfkfb3*	.0073	-5.71	TC0200002961.mm.2
*Clcn5*	4.01E-05	-5.64	TC0X00001766.mm.2
*Gm11127*	.0003	-5.59	TC1700001996.mm.2
*Paqr9*	.0002	-5.55	TC0900001195.mm.2
*Grina*	9.57E-06	-5.51	TC1500000580.mm.2
*Atp11a*	7.34E-05	-5.51	TC0800000111.mm.2
*Tmem229b*	.0016	-5.35	TC1200001974.mm.2
*Pdzk1*	.0003	-5.25	TC0300000958.mm.2
*Gpr50*	4.24E-07	-5.18	TC0X00000653.mm.2
*Gulp1*	3.29E-06	-5.13	TC0100000338.mm.2
*S100a1*	.0007	-5.08	TC0300002374.mm.2
*Cd36*	.0314	-5.07	TC0500001992.mm.2
*Herc6*	.0068	-4.92	TC0600003503.mm.2
*Ctsa*	1.37E-05	-4.89	TC0200002537.mm.2
*Lama3*	.0053	-4.82	TC1800000103.mm.2
*Uap1l1*	1.24E-05	-4.75	TC0200003147.mm.2
*Hexa*	1.35E-05	-4.71	TC0900000748.mm.2
*Pxdc1*	.0008	-4.67	TC1300001789.mm.2
*Pdzk1ip1*	4.56E-05	-4.65	TC0400001217.mm.2
*Ace*	.0486	-4.65	TC1100001767.mm.2
*Spns2*	.0002	-4.61	TC1100003146.mm.2
*Ifit1*	.0002	-4.6	TC1900000504.mm.2
*Tmem144*	7.34E-05	-4.55	TC0300002203.mm.2
*Snx8*	.0021	-4.52	TC0500003507.mm.2
*Lbh*	6.50E-05	-4.45	TC1700001147.mm.2
*Ctss*	1.84E-06	-4.44	TC0300000903.mm.2
*Plpp3*	4.67E-05	-4.41	TC0400001091.mm.2
*Plagl1*	.0001	-4.41	TC1000000093.mm.2
*Fam198b*	8.59E-05	-4.36	TC0300000627.mm.2
*H6pd*	.0008	-4.3	TC0400004058.mm.2
*Entpd2*	7.86E-06	-4.27	TC0200005462.mm.2
*Gm16404*	.027	-4.27	TC0X00000505.mm.2
*Naga*	.0001	-4.25	TC1500001942.mm.2
*Gm10721*	.0085	-4.24	TC0900000003.mm.2
*Kazn*	1.38E-06	-4.23	TC0400003900.mm.2
*Ppp1r32*	4.72E-06	-4.19	TC1900001102.mm.2
*Cyp3a41a*	.0076	-4.19	TC0500003745.mm.2
*Rab3il1*	.0012	-4.18	TC1900000184.mm.2
*Mcoln1*	4.92E-06	-4.15	TC0800000010.mm.2
*Espn*	.0165	-4.15	TC0400004090.mm.2
*Fbp1*	1.48E-05	-4.11	TC1300002176.mm.2
*Gba*	7.23E-06	-4.1	TC0300000750.mm.2
*Acot5*	.0019	-4.07	TC1200000801.mm.2
*Gm8615*	.0001	-4.06	TC0500003651.mm.2
*Cyp3a41b*	.006	-4.02	TC0500003744.mm.2
*Arnt2*	.0002	-4	TC0700003652.mm.2
*Thsd4*	9.49E-05	-3.95	TC0900002428.mm.2
*Vsig10l*	4.22E-05	-3.94	TSUnmapped00000002.mm.2
*Gpr151*	.0058	-3.92	TC1800001259.mm.2
*Mfsd12*	.0001	-3.89	TC1000000895.mm.2
*Pi4k2a*	1.08E-05	-3.8	TC1900000609.mm.2
*Mfsd7c*	.0007	-3.78	TC1200000839.mm.2
*Cox6b2*	.0003	-3.76	TC0700002169.mm.2
*Nabp1*	.0005	-3.73	TC0100002305.mm.2
*Arsa*	5.41E-05	-3.72	TC1500002056.mm.2
*Ros1*	3.08E-05	-3.71	TC1000002154.mm.2
*Tfec*	.004	-3.71	TC0600001890.mm.2
*Gatsl2*	.0011	-3.7	TC0500001546.mm.2
*Mamdc4*	.0121	-3.69	TC0200005493.mm.2
*Cd55*	.0018	-3.68	TC0100003037.mm.2
*Npc1l1*	.0084	-3.66	TC1100002165.mm.2
*Rragd*	.0005	-3.65	TC0400000275.mm.2
*1700049E17Rik2*	.048	-3.64	TC1400000418.mm.2
*Rnd2*	.0002	-3.63	TC1100001658.mm.2
*Folr1*	.0076	-3.63	TC0700003846.mm.2
*Naglu*	4.29E-05	-3.62	TC1100001638.mm.2
*Tmppe; Glb1*	1.61E-05	-3.61	TC0900001513.mm.2
*Gm13178*	.0001	-3.6	TC0400003951.mm.2
*Ephx2*	.0017	-3.6	TC1400002266.mm.2
*Pqlc2*	.0001	-3.59	TC0400003847.mm.2
*Trim5*	.0005	-3.59	TC0700003936.mm.2
*Gga2*	1.24E-06	-3.55	TC0700004221.mm.2
*Amn*	1.25E-05	-3.55	TC1200001233.mm.2
*Trim12a*	.0004	-3.54	TC0700003938.mm.2
*Gnpda1*	.0005	-3.53	TC1800001230.mm.2
*Tpp1*	1.79E-05	-3.52	TC0700003990.mm.2
*Gm438*	2.42E-05	-3.5	TC0400003953.mm.2
*Smcp*	.0003	-3.5	TC0300002397.mm.2
*Adora2b*	.0002	-3.49	TC1100000792.mm.2
*Flcn*	2.33E-05	-3.48	TC1100002862.mm.2
*Cdhr2*	.004	-3.48	TC1300000635.mm.2
*Igsf23*	.0002	-3.44	TC0700002509.mm.2
*Gm21738*	.009	-3.43	TC1400001514.mm.2
*Gpr157*	.0002	-3.42	TC0400001969.mm.2
*Clec2e*	.0357	-3.41	TC0600003225.mm.2
*Abca14*	6.77E-05	-3.38	TC0700001725.mm.2
*Galc*	.0001	-3.37	TC1200002178.mm.2
*Gm14625*	.0494	-3.36	TC0X00000504.mm.2
*Dct*	1.01E-05	-3.35	TC1400002709.mm.2
*Gsta3*	.0002	-3.35	TC0100000135.mm.2
*Fhl1*	.0165	-3.32	TC0X00000525.mm.2
*Anxa5*	.0002	-3.3	TC0300001850.mm.2
*Cidec*	.0072	-3.3	TC0600002920.mm.2
*Sema6a*	.0106	-3.3	TC1800001312.mm.2
*Ifit1bl2*	.026	-3.3	TC1900001423.mm.2
*Glmp*	5.68E-05	-3.28	TC0300000722.mm.2
*Podxl*	.0009	-3.26	TC0600002020.mm.2
*Sectm1b*	.0308	-3.26	TC1100004241.mm.2
*Adm*	.0004	-3.24	TC0700001630.mm.2
*Rora*	1.78E-05	-3.23	TSUnmapped00000043.mm.2
*Slc38a6*	.0003	-3.21	TC1200000641.mm.2
*Casp4*	.0006	-3.21	TC0900000032.mm.2
*Gatm*	8.10E-07	-3.2	TC0200004511.mm.2
*Mettl7b*	.0004	-3.2	TC1000003169.mm.2
*Mafb*	.0005	-3.2	TC0200005052.mm.2
*Clca4b*	.0485	-3.19	TC0300003082.mm.2
*Abcg8*	.0014	-3.18	TC1700001265.mm.2
*Ddit3*	.0006	-3.16	TSUnmapped00000016.mm.2
*Slc17a5*	.0004	-3.15	TC0900002728.mm.2
*Lgals3bp*	3.70E-06	-3.14	TC1100004159.mm.2
*Acss1*	.0007	-3.13	TC0200004843.mm.2
*Gm10715*	.0059	-3.12	TC0900000008.mm.2
*Hist1h1c*	.0003	-3.11	TC1300000258.mm.2
*Trim30d*	.0116	-3.11	TC0700003946.mm.2
*Rab31*	.0006	-3.09	TC1700002414.mm.2
*Nox4*	.0004	-3.07	TC0700001252.mm.2
*Dgat2*	.004	-3.07	TC0700003793.mm.2
*Tm6sf2*	.0011	-3.06	TC0800000734.mm.2
*Ypel5*	.0046	-3.06	TC1700001145.mm.2
*Scpep1*	.0002	-3.05	TC1100003506.mm.2
*Tapbp*	.0013	-3.03	TC1700002778.mm.2
*Gm20806*	.0376	-3.03	TC0Y00000079.mm.2
*Cstb*	9.32E-05	-3.02	TC1000000784.mm.2
*Gm5431*	.0028	-3.02	TC1100002642.mm.2
*Ret*	6.84E-05	-3.01	TC0600002996.mm.2
*Ctso*	1.42E-06	-2.98	TC0300000635.mm.2
*Syne1*	.0003	-2.97	TC1000001679.mm.2
*Slc39a4*	.0004	-2.97	TC1500001783.mm.2
*Rhbdl2*	.0056	-2.96	TC0400001405.mm.2
*Sgk3*	4.65E-05	-2.95	TC0100000044.mm.2
*Stat2*	.0028	-2.95	TC1000001596.mm.2
*Lipe*	.0021	-2.94	TC0700002632.mm.2
*Gbp7*	.0001	-2.93	TC0300001445.mm.2
*Amdhd2*	.0002	-2.92	TC1700001618.mm.2
*Abcc2*	.0006	-2.92	TC1900000636.mm.2
*Sugct*	4.89E-05	-2.91	TC1300001494.mm.2
*Adamtsl5*	.001	-2.91	TC1000002497.mm.2
*Oasl2*	.0103	-2.91	TC0500001239.mm.2
*Usp54*	7.06E-05	-2.88	TC1400001544.mm.2
*Creb3l3*	.0005	-2.88	TC1000002525.mm.2
*Cdipt*	.0001	-2.87	TC0700001827.mm.2
*Prkg2*	.0002	-2.87	TC0500002814.mm.2
*Ceacam20*	.029	-2.87	TC0700000315.mm.2
*Slc35e3*	.0006	-2.86	TC1000002943.mm.2
*Slc6a8*	.0089	-2.86	TC0X00000690.mm.2
*Slc23a1*	.0024	-2.84	TC1800001175.mm.2
*Slc11a2*	.0074	-2.84	TC1500002238.mm.2
*Prdm1*	.014	-2.84	TC1000002099.mm.2
*Ano10*	1.81E-05	-2.82	TC0900003282.mm.2
*Trim34b; Trim34a*	.0004	-2.82	TC0700001520.mm.2
*Smim1*	3.34E-06	-2.8	TC0400004112.mm.2
*Depdc7*	.0002	-2.8	TC0200004249.mm.2
*Aldh7a1*	2.06E-05	-2.79	TC1800001366.mm.2
*Ms4a10*	.0005	-2.79	TC1900001119.mm.2
*Wdr81*	7.61E-06	-2.78	TC1100003225.mm.2
*Slc9a6*	.0003	-2.78	TC0X00000522.mm.2
*Gusb*	.0003	-2.78	TC0500003313.mm.2
*Slc18a1*	.0072	-2.78	TC0800002357.mm.2
*Plekhg6*	.0309	-2.76	TC0600003146.mm.2
*Morn4*	.0001	-2.75	TC1900001519.mm.2
*Mfge8*	.0001	-2.75	TC0700003552.mm.2
*Gml2*	.0023	-2.75	TC1500000545.mm.2
*Fbxo6*	.0006	-2.74	TC0400004014.mm.2
*Serpinb6b*	.0155	-2.74	TC1300000362.mm.2
*Slc2a2*	.027	-2.74	TC0300000180.mm.2
*Lect2*	.002	-2.72	TC1300002083.mm.2
*Hcls1*	6.39E-05	-2.71	TC1600000529.mm.2
*Ctsz*	2.79E-05	-2.7	TC0200005314.mm.2
*Akr1c19*	.0003	-2.7	TC1300000020.mm.2
*Man2b1*	4.71E-05	-2.69	TC0800000983.mm.2
*Bst2*	.0015	-2.69	TC0800002431.mm.2
*Ugdh*	.0007	-2.68	TC0500002486.mm.2
*Hgd*	.001	-2.68	TC1600000534.mm.2
*Gpr137b-ps*	.0001	-2.66	TC1300001451.mm.2
*Lrrn1*	.0003	-2.66	TC0600001189.mm.2
*Agpat3*	1.21E-05	-2.65	TC1000002448.mm.2
*Snx32*	.0003	-2.65	TC1900000971.mm.2
*Vmn1r116*	.0011	-2.65	TC0700000325.mm.2
*Optn*	.005	-2.65	TC0200002872.mm.2
*Slc46a1*	.0302	-2.65	TC1100001161.mm.2
*Igfals*	.0002	-2.64	TC1700000355.mm.2
*Hexb*	.0005	-2.64	TC1300002481.mm.2
*Trim12c*	.0016	-2.64	TC0700003940.mm.2
*Aoah*	4.87E-05	-2.63	TC1300000157.mm.2
*Shisa5*	.0001	-2.63	TC0900001431.mm.2
*Stra6l*	.0001	-2.63	TC0400000496.mm.2
*Fndc5*	.0008	-2.63	TC0400001514.mm.2
*Ncoa4*	.0015	-2.63	TC1400000340.mm.2
*Gm6367*	.005	-2.63	TC5_JH584299_random00000006.mm.2
*Ddit3*	.0025	-2.62	TC1000001557.mm.2
*Apoc3*	.0485	-2.62	TC0900002218.mm.2
*Ppfibp2*	2.14E-05	-2.61	TC0700001581.mm.2
*Irf9*	.0034	-2.6	TC1400002834.mm.2
*Rbp2*	.0313	-2.6	TC0900001234.mm.2
*Nipa2*	.0001	-2.59	TC0700003100.mm.2
*E330020D12Rik*	.0005	-2.59	TC0100003276.mm.2
*Hif3a*	.002	-2.59	TC0700002445.mm.2
*Lpar3*	.003	-2.59	TC0300001490.mm.2
*Stk10*	.0058	-2.59	TC1100000343.mm.2
*Nckap5*	.0471	-2.59	TC0100002998.mm.2
*Fam78a*	.0029	-2.58	TC0200005496.mm.2
*Apoa4*	.0031	-2.58	TC0900000560.mm.2
*Slc26a11*	.0001	-2.57	TC1100002001.mm.2
*Gm6034*	.0053	-2.57	TC1700000719.mm.2
*Vmn2r47*	.0281	-2.57	TC0700002249.mm.2
*Rgl1*	.0004	-2.56	TC0100003264.mm.2
*Clcn7*	5.83E-05	-2.55	TC1700000362.mm.2
*Sesn2*	.0001	-2.55	TC0400003674.mm.2
*Aplp1*	.0125	-2.55	TC0700002759.mm.2
*Smpdl3a*	.0029	-2.54	TC1000000509.mm.2
*Gal3st2*	.0482	-2.54	TC0100000948.mm.2
*Gm766*	.0393	-2.53	TC0600003382.mm.2
Tmem104	.0004	-2.52	TC1100001887.mm.2
*Wsb2*	.0006	-2.52	TC0500001297.mm.2
*Gm20917*	.0293	-2.52	TC0Y00000487.mm.2
*Ctns*	.0001	-2.51	TC1100003159.mm.2
*Ajuba*	.0011	-2.51	TC1400002059.mm.2
*Btnl2*	.0017	-2.51	TC1700000629.mm.2
*Gaa*	2.93E-05	-2.5	TC1100001999.mm.2
*Cxxc5*	7.59E-05	-2.5	TC1800000319.mm.2
*G6pc*	.0015	-2.5	TC1100001654.mm.2
*Ctsh*	.0002	-2.49	TC0900001155.mm.2
*Ggh*	3.09E-05	-2.48	TC0400000169.mm.2
*Dirc2*	.0015	-2.48	TC1600001553.mm.2
*Grn*	5.77E-05	-2.47	TC1100001688.mm.2
*Tsc1*	.0002	-2.47	TC0200000490.mm.2
*Ctsd; Ifitm10*	1.58E-05	-2.46	TC0700004565.mm.2
*Tmem51*	5.29E-05	-2.46	TC0400003897.mm.2
*Xkr9*	.0016	-2.46	TC0100000071.mm.2
*Lrat*	.0017	-2.45	TC0300002229.mm.2
*Cachd1*	.0002	-2.44	TC0400001020.mm.2
*Bbs9*	.0002	-2.44	TC0900000223.mm.2
*Gla*	.0005	-2.44	TC0X00002995.mm.2
*3110057O12Rik;* *Gm2011*	.0016	-2.44	TC0300000309.mm.2
*Atp6v1d*	.0006	-2.43	TC1200001971.mm.2
*Glyat*	.0018	-2.43	TC1900000250.mm.2
*3830403N18Rik*	.016	-2.43	TC0X00000512.mm.2
*Gm4216; Gm4177*	.0311	-2.43	TC0700002547.mm.2
*Gm4177; Gm4216*	.0311	-2.43	TC0700002576.mm.2
*Fuca2*	4.66E-06	-2.42	TC1000000095.mm.2
*Ifnar2*	.0001	-2.42	TC1600002172.mm.2
*Serinc5*	.0002	-2.42	TC1300001036.mm.2
*Dpp7*	.0034	-2.41	TC0200003146.mm.2
*Tmem37*	6.65E-05	-2.4	TC0100002963.mm.2
*Bmp1*	.0005	-2.4	TC1400002343.mm.2
*Asah2*	.0445	-2.4	TC1900001388.mm.2
*Lrrc8a*	.0001	-2.39	TC0200005466.mm.2
*Tdrp*	.0003	-2.39	TC0800001748.mm.2
*Atp6v0a1*	.0022	-2.39	TC1100001634.mm.2
*Mif4gd*	1.11E-05	-2.38	TC1100004299.mm.2
*Creg1*	3.64E-05	-2.38	TC0100001526.mm.2
*Slc25a44*	.0003	-2.38	TC0300002311.mm.2
*Wbp2*	.0005	-2.38	TC1100004102.mm.2
*Plk3*	.0091	-2.38	TC0400003361.mm.2
*Cpne8*	.0142	-2.38	TC1500002067.mm.2
*Parp10*	3.97E-05	-2.37	TC1500002347.mm.2
*Rbpms2*	5.30E-05	-2.36	TC0900000848.mm.2
*Cln6*	.0001	-2.36	TC0900000793.mm.2
*Tpcn1*	.0004	-2.36	TC0500003169.mm.2
*Acsl1*	.0004	-2.36	TC0800000538.mm.2
*Slc35f6; Mir5625*	.0222	-2.36	TC0500000297.mm.2
*Tmem184a*	.0001	-2.35	TC0500003742.mm.2
*Cln3*	.0021	-2.35	TC0700004285.mm.2
*Aldh2*	.0025	-2.34	TC0500003193.mm.2
*Isg15*	.0402	-2.34	TC0400004171.mm.2
*Ifi27*	.0004	-2.33	TC1200001030.mm.2
*Car5a*	.0018	-2.33	TC0800003060.mm.2
*Slc6a19*	.0075	-2.33	TC1300002303.mm.2
*Cyp3a25*	.0317	-2.33	TC0500003601.mm.2
*Bcr*	.0006	-2.32	TC1000000708.mm.2
*Olfr165*	.0372	-2.32	TC1600001328.mm.2
*Enpp5*	1.36E-05	-2.31	TC1700000842.mm.2
*Rras*	.0004	-2.31	TC0700000826.mm.2
*Plekhg5*	.0009	-2.31	TC0400002011.mm.2
*Cpeb4*	.0014	-2.31	TC1100000324.mm.2
*Piga*	.0026	-2.31	TC0X00001668.mm.2
*Npas2*	.0185	-2.31	TC0100000282.mm.2
*9230113P08Rik*	.0227	-2.31	TC0900000342.mm.2
*Cpn1*	3.73E-06	-2.3	TC1900001549.mm.2
*Capn15*	9.83E-05	-2.3	TC1700001684.mm.2
*Snx30*	.0004	-2.3	TC0400000662.mm.2
*Rcan1*	.0004	-2.3	TC1600002094.mm.2
*Tmem106a*	.0008	-2.3	TC1100001661.mm.2
*Cystm1*	.001	-2.3	TC1800000327.mm.2
*Hebp1*	.0001	-2.29	TC0600003314.mm.2
*Sdr16c5*	.0005	-2.29	TC0400002136.mm.2
*Pyroxd2*	.0008	-2.29	TC1900001529.mm.2
*Sult1d1*	.0191	-2.29	TC0500002699.mm.2
*Kif12*	.0192	-2.29	TC0400002797.mm.2
*Pnpla7*	6.98E-05	-2.28	TC0200000393.mm.2
*Npc2*	.0001	-2.28	TC1200002081.mm.2
*Gabarapl2*	.0005	-2.28	TC0800001341.mm.2
*Spg20*	.0081	-2.28	TC0300000416.mm.2
*Cfi*	.0002	-2.27	TC0300001313.mm.2
*Ttbk2*	.0009	-2.27	TC0200005502.mm.2
*Hpgds*	.0009	-2.27	TC0600002404.mm.2
*Dnase1l1*	.0016	-2.27	TC0X00002448.mm.2
*Arhgap26*	.014	-2.27	TC1800001744.mm.2
*Ttc7b*	1.88E-05	-2.26	TC1200002224.mm.2
*Atp6v1b2*	9.19E-05	-2.26	TC0800000711.mm.2
*Mir692-3; Ftl1;* *Ftl2-ps*	.0003	-2.26	TC0400001446.mm.2
*Ttr*	.0004	-2.26	TC1800000158.mm.2
*Gss*	.0013	-2.26	TC0200004965.mm.2
*Nxpe3*	.0043	-2.26	TC1600001743.mm.2
*Slc52a3*	.0236	-2.26	TC0200002272.mm.2
*Ldlrad4*	.02E-05	-2.25	TC1800000714.mm.2
*Ppp1r21*	6.79E-05	-2.25	TC1700001322.mm.2
*Cryz*	.0008	-2.25	TC0300001554.mm.2
*Slc5a9*	.0028	-2.25	TC0400003285.mm.2
*Slc7a9*	.0164	-2.25	TC0700000661.mm.2
*Sirt2*	1.60E-05	-2.24	TC0700000511.mm.2
*Lrp12*	.0025	-2.24	TC1500001431.mm.2
*Ank3*	.043	-2.24	TC1000000661.mm.2
*Dcst2*	.0003	-2.22	TC0300000761.mm.2
*Gsdmd*	.0005	-2.22	TC1500000565.mm.2
*Pdgfc*	.0051	-2.22	TC0300000631.mm.2
*Acadl*	6.39E-06	-2.21	TC0100002495.mm.2
*Tmem106b*	.001	-2.21	TC0600000091.mm.2
*Il4ra*	.0014	-2.21	TC0700001793.mm.2
*Kif21b*	.0024	-2.21	TC0100001262.mm.2
*Gm7030*	.0058	-2.21	TC1700002821.mm.2
*Tram2*	.0033	-2.2	TC0100002038.mm.2
*Gm10801*	.0081	-2.2	TC0200001502.mm.2
*Mfhas1*	.0365	-2.2	TC0800000426.mm.2
*Tmc5*	.0015	-2.19	TC0700001698.mm.2
*Stx7*	.0051	-2.19	TC1000000208.mm.2
*Abhd3*	.009	-2.19	TC1800000980.mm.2
*Mup12*	.0168	-2.19	TC0400004212.mm.2
*Chp2*	.0493	-2.19	TC0700001754.mm.2
*Sfxn3*	9.54E-06	-2.18	TC1900000664.mm.2
*Fuca1*	6.73E-05	-2.18	TC0400001668.mm.2
*Slc37a4*	.0005	-2.18	TC0900000509.mm.2
*Uba7*	.0017	-2.18	TC0900003335.mm.2
*Tcn2*	.0022	-2.18	TC1100002103.mm.2
*Gch1*	.0046	-2.18	TC1400001930.mm.2
*Apol9a*	.028	-2.18	TC1500001809.mm.2
*Vmn1r143;* *Vmn1r95*	.0373	-2.18	TC0700000318.mm.2
*Trf*	.0006	-2.17	TC0900003344.mm.2
*Sat2*	.0035	-2.17	TC1100000917.mm.2
*Dhx58*	.006	-2.17	TC1100003779.mm.2
*Ccdc152*	.0077	-2.17	TC1500001135.mm.2
*Pcyt1a*	.0077	-2.17	TC1600000443.mm.2
*Leap2*	.0208	-2.17	TC1100002728.mm.2
*Arrdc4*	1.94E-05	-2.16	TC0700003464.mm.2
*Coro7*	.0006	-2.16	TC1600001134.mm.2
*Arsg*	.0009	-2.16	TC1100001832.mm.2
*Slc7a7*	.0012	-2.16	TC1400002054.mm.2
*H2-Q1*	.0126	-2.16	TC1700002791.mm.2
*Tom1l2*	.023	-2.16	TC1100002871.mm.2
*Wdfy3*	.0001	-2.14	TC0500002859.mm.2
*Ube2l6*	.0055	-2.14	TC0200001222.mm.2
*Ept1*	.0002	-2.13	TC0500000289.mm.2
*Lrrfip1*	.0039	-2.13	TC0100000884.mm.2
*Crebrf*	.0059	-2.13	TC1700000419.mm.2
*Sema7a*	.0289	-2.13	TC0900000720.mm.2
*Gm20917*	.0363	-2.13	TC0Y00000061.mm.2
*Prcp*	3.88E-05	-2.12	TC0700001321.mm.2
*Trpc3*	.0136	-2.12	TC0300001858.mm.2
*Bcas3*	.0259	-2.12	TC1100001293.mm.2
*Hsd17b13*	.0001	-2.11	TC0500002881.mm.2
*Aim1l*	.0008	-2.11	TC0400001626.mm.2
*Znfx1*	.0029	-2.11	TC0200005181.mm.2
*Gpr137b*	.0002	-2.1	TC1300001461.mm.2
*Grhpr*	.0011	-2.1	TC0400000481.mm.2
*Susd1*	.0015	-2.1	TC0400002755.mm.2
*Proz*	.0016	-2.1	TC0800000117.mm.2
*Rnf123*	.0017	-2.1	TC0900003050.mm.2
*Dnal1*	.005	-2.1	TC1200000803.mm.2
*Slc43a2*	.0162	-2.1	TC1100001072.mm.2
*Plbd2*	.0001	-2.09	TC0500003168.mm.2
*Dapp1*	.0014	-2.09	TC0300003013.mm.2
*Itgb6*	.0044	-2.09	TC0200003605.mm.2
*Spg21*	.0001	-2.08	TC0900000845.mm.2
*Rab24*	.0002	-2.08	TC1300002052.mm.2
*Hmgcl*	.0003	-2.08	TC0400001670.mm.2
*Aph1b*	.0008	-2.08	TC0900002529.mm.2
*Ormdl3*	.0017	-2.08	TC1100003683.mm.2
*Gm8050*	.0029	-2.08	TC1400001415.mm.2
*Oas1g*	.0291	-2.08	TC0500003180.mm.2
*Pbld1*	.0295	-2.08	TC1000000596.mm.2
*Rab17*	.0006	-2.07	TC0100002793.mm.2
*Fam46a*	.0128	-2.07	TC0900002770.mm.2
*Sec24d*	.0183	-2.07	TC0300001255.mm.2
*Gm10324*	.0272	-2.07	TC1300000803.mm.2
*Tifa*	.0002	-2.06	TC0300001292.mm.2
*Il18r1*	.0049	-2.06	TC0100000299.mm.2
*Gabarapl1*	.0146	-2.06	TC0600001536.mm.2
*Sord*	9.59E-05	-2.05	TC0200001843.mm.2
*6330416G13Rik*	.0004	-2.05	TC0400000726.mm.2
*Soat2*	.0033	-2.05	TC1500001090.mm.2
*Plekhn1*	.0052	-2.05	TC0400004173.mm.2
*Pla2g12b*	.0253	-2.05	TC1000000534.mm.2
*Fibp*	.0001	-2.04	TC1900000069.mm.2
*Acp5*	.0032	-2.04	TC0900001900.mm.2
*Slc34a2*	.0075	-2.04	TC0500000536.mm.2
*Arfgap3*	.023	-2.04	TC1500001971.mm.2
*Sidt2*	2.32E-05	-2.03	TC0900002213.mm.2
*Mospd1*	.0003	-2.03	TC0X00002238.mm.2
*Rab8b*	.0006	-2.03	TC0900002533.mm.2
*Lmbrd1*	.0006	-2.03	TC0100000171.mm.2
*Rnf213*	.0012	-2.03	TC1100002003.mm.2
*Arhgef3*	.0032	-2.03	TC1400000271.mm.2
*Nudt16l1*	.0044	-2.03	TC1600000040.mm.2
*Cyp3a16*	.0071	-2.03	TC0500003596.mm.2
*Tnfrsf12a*	.0095	-2.03	TC1700001598.mm.2
*Eps8l3*	.019	-2.03	TC0300001123.mm.2
*Emp2*	.0301	-2.03	TC1600001191.mm.2
*Ctsb*	7.39E-05	-2.02	TC1400000895.mm.2
*Unc119*	.0003	-2.02	TC1100001158.mm.2
*Slc5a6*	.0014	-2.02	TC0500002170.mm.2
*Plod1*	.0261	-2.02	TC0400004004.mm.2
*Plin3*	.0006	-2.01	TC1700002321.mm.2
*Kifc3*	.0009	-2.01	TC0800002739.mm.2
*Folr2*	.002	-2.01	TC0700003845.mm.2
*Slc22a5*	.003	-2.01	TC1100002748.mm.2
*Tollip*	.0069	-2.01	TC0700004548.mm.2
*Gm11168*	.0074	-2.01	TC0900000002.mm.2
*F2rl1*	.0002	-2	TC1300002460.mm.2
*Mfsd7b*	.001	-2	TC0100003797.mm.2
*Tbc1d1*	.0015	-2	TC0500000591.mm.2
*Dennd5b*	.0023	-2	TC0600003475.mm.2
*Itgb5*	.0198	-2	TC1600000480.mm.2
*Golm1*	.0006	2	TC1300002119.mm.2
*Atp5e*	.0042	2	TC0200005316.mm.2
*Hk2*	.0066	2	TC0600002550.mm.2
*Cyp51*	.0002	2.01	TC0500001894.mm.2
*Slc25a37*	.003	2.01	TC1400002310.mm.2
*Pprc1*	.0062	2.01	TC1900000681.mm.2
*Cdca8*	.0174	2.01	TC0400003516.mm.2
*Aurkb*	.0493	2.01	TC1100000892.mm.2
*Ttc13*	.0005	2.02	TC0800003173.mm.2
*Actg1; Mir6935*	.0065	2.02	TC1100004202.mm.2
*Dbf4*	.0267	2.02	TC0500001931.mm.2
*Ctdspl2*	.0011	2.03	TC0200001829.mm.2
*Mcm7; Mir93;* *Mir25*	.021	2.03	TC0500003472.mm.2
*Nifk*	.0217	2.03	TC0100001058.mm.2
*Nr1d2*	8.77E-05	2.04	TC1400001499.mm.2
*2810004N23Rik*	.0012	2.04	TC0800003176.mm.2
*E2f2*	.0016	2.04	TC0400001678.mm.2
*Dynll1*	.0016	2.04	TC0500003095.mm.2
*Mex3c*	.0021	2.04	TC1800000771.mm.2
*Tor3a*	.0082	2.04	TC0100003320.mm.2
*Slfn9*	.0102	2.04	TC1100003379.mm.2
*Lgals12*	.0456	2.04	TC1900001051.mm.2
*Cep78*	.003	2.05	TC1900001205.mm.2
*Spib*	.0044	2.05	TC0700002967.mm.2
*Orc2*	.0048	2.05	TC0100002383.mm.2
*Smim6*	.0149	2.05	TC1100001912.mm.2
*Flnb*	.0002	2.06	TC1400000071.mm.2
*Fam183b*	.03	2.06	TC1100002834.mm.2
*Cttnbp2nl*	6.71E-05	2.07	TC0300002665.mm.2
*Eef2*	.001	2.07	TC1000000884.mm.2
*Rap2a*	.0014	2.07	TC1400001306.mm.2
*Gtpbp10*	.0018	2.08	TC0500001910.mm.2
*Ccne1*	.0267	2.08	TC0700002876.mm.2
*Mdc1*	.0463	2.09	TC1700000705.mm.2
*Scg3*	.0105	2.1	TC0900002681.mm.2
*Il1rap*	.0148	2.1	TC1600000376.mm.2
*Nt5c3b*	.0097	2.11	TC1100003771.mm.2
*Tlr3*	.0001	2.12	TC0800002146.mm.2
*Tmem117*	.0009	2.12	TC1500000933.mm.2
*Atp2a3*	.0221	2.12	TC1100001010.mm.2
*Gse1*	.0222	2.12	TC0800001437.mm.2
*Sh3bgrl*	.0003	2.13	TC0X00003399.mm.2
*Avil*	.0116	2.13	TC1000001543.mm.2
*Fam83c*	.0148	2.13	TC0200004974.mm.2
*Mme*	.002	2.14	TC0300000500.mm.2
*Kctd12; Mir5130*	.0175	2.14	TC1400002632.mm.2
*Trp53bp1*	.0006	2.16	TC0200004482.mm.2
*Ppp2r3d*	.0039	2.16	TC0900003321.mm.2
*Asf1b*	.0096	2.16	TC0800000937.mm.2
*Slitrk6*	9.84E-05	2.17	TC1400002689.mm.2
*Prss30*	.0001	2.17	TC1700001611.mm.2
*Sipa1l1*	.0004	2.17	TC1200000769.mm.2
*Acsm3*	.0037	2.17	TC0700001715.mm.2
*Igsf9b*	.0043	2.17	TC0900000259.mm.2
*Slc41a2*	.014	2.17	TC1000002580.mm.2
*Tex12*	.0396	2.17	TC0900002270.mm.2
*Zak*	.0026	2.18	TC0200001053.mm.2
*Strip2*	.0125	2.18	TC0600000250.mm.2
*Hpgd*	.0459	2.18	TC0800000621.mm.2
*Cdc42bpa*	.0005	2.19	TC0100001703.mm.2
*Lyz1*	.0383	2.19	TC1000002930.mm.2
*Nusap1*	.0237	2.2	TC0200001776.mm.2
*AI506816*	.0495	2.21	TC0500002046.mm.2
*Rangrf*	.0008	2.22	TC1100003024.mm.2
*Rab27b*	.0026	2.22	TC1800001535.mm.2
*Eif4g2*	.0057	2.22	TC0700004108.mm.2
*Smpx*	.0081	2.22	TC0X00001601.mm.2
*Sytl2*	.0383	2.22	TC0700001280.mm.2
*Megf9*	.0012	2.23	TC0400002849.mm.2
*Impdh1*	.0253	2.23	TC0600001974.mm.2
*Reg3g*	2.93E-05	2.24	TC0600002539.mm.2
*Epb41l2*	9.70E-05	2.24	TC1000000224.mm.2
*Otud6b*	.0241	2.24	TC0400002236.mm.2
*Atxn7l1*	.0021	2.26	TC1200000287.mm.2
*Klk1*	.0371	2.26	TC0700000796.mm.2
*Hpd*	.0459	2.27	TC0500003227.mm.2
*Hoxb9*	.0084	2.28	TC1100001492.mm.2
*Myof*	.0087	2.28	TC1900001461.mm.2
*Ptpn22*	.0088	2.28	TC0300001064.mm.2
*Mpnd*	.0032	2.29	TC1700000996.mm.2
*Pla2g4a*	.0054	2.3	TC0100003233.mm.2
*Pbk*	.017	2.3	TC1400000940.mm.2
*Lrig1*	.0479	2.3	TC0600002773.mm.2
*Rab27a*	.0176	2.31	TC0900000989.mm.2
*Mid2*	.0002	2.32	TC0X00001383.mm.2
*Gjb3*	.0079	2.33	TC0400003566.mm.2
*Prr11*	.0038	2.34	TC1100003470.mm.2
*Inpp5d*	.0149	2.35	TC0100000822.mm.2
*Slc2a1*	.0042	2.38	TC0400001325.mm.2
*Plce1*	1.11E-05	2.4	TC1900000552.mm.2
*5330417C22Rik*	.0226	2.4	TC0300002743.mm.2
*Fxyd3*	.0235	2.4	TC0700002789.mm.2
*Ces1f*	.0459	2.4	TC0800002705.mm.2
*Gale*	.0005	2.41	TC0400001671.mm.2
*Creb3l1*	.0174	2.41	TC0200004133.mm.2
*Spire1*	.0009	2.42	TC1800001507.mm.2
*Gm17482*	.0016	2.42	TC0600001278.mm.2
*Apobec3*	.0016	2.43	TC1500000688.mm.2
*Ccnd1; Mir3962*	.0027	2.43	TC0700004610.mm.2
*Adcy8*	.0011	2.44	TC1500001641.mm.2
*Arhgef38*	.0023	2.44	TC0300002977.mm.2
*Ifitm3*	.0056	2.44	TC0700004515.mm.2
*Cyp4f40*	.0011	2.46	TC1700000566.mm.2
*Oit1*	.0408	2.5	TC1400001440.mm.2
*Siglecf*	.0086	2.51	TC0700000756.mm.2
*Gdpd1*	3.15E-06	2.52	TC1100003466.mm.2
*Myo5c*	.0009	2.53	TC0900001011.mm.2
*Hist1h1d*	.0031	2.54	TC1300000248.mm.2
*Agr2*	.0096	2.55	TC1200000316.mm.2
*Syt7*	.0119	2.55	TC1900000197.mm.2
*Prmt3*	.0173	2.56	TC0700000904.mm.2
*Gm10935*	.0119	2.57	TC0700002687.mm.2
*Asns*	.0315	2.58	TC0600001839.mm.2
*Fut8*	.0021	2.59	TC1200000688.mm.2
*Pcsk1*	.0109	2.62	TC1300000908.mm.2
*Hepacam2*	.0219	2.62	TC0600001788.mm.2
*Serpina1d*	.0289	2.64	TC1200002532.mm.2
*Zfp367*	.0024	2.65	TC1300002197.mm.2
*Dlgap5*	.0035	2.65	TC1400001935.mm.2
*Adh1*	.0042	2.68	TC0300001409.mm.2
*Efnb2*	.0005	2.7	TC0800001654.mm.2
*Pou2f3*	.0079	2.7	TC0900002127.mm.2
*Gsta4*	.0082	2.7	TC0900001048.mm.2
*Ntn4*	.0039	2.71	TC1000001138.mm.2
*Serpina1b*	.0439	2.73	TC1200002531.mm.2
*Cd44*	.0003	2.74	TC0200004219.mm.2
*Gnat3*	.0016	2.8	TC0500000134.mm.2
*Rbp7*	.002	2.82	TC0400004041.mm.2
*Tmprss2*	.0038	2.9	TC1600002152.mm.2
*Rap1gap*	.0204	2.94	TC0400001712.mm.2
*Nfix*	2.98E-06	2.95	TC0800002589.mm.2
*Reg3b*	.0044	2.97	TC0600000875.mm.2
*Arg2*	.0098	2.98	TC1200000715.mm.2
*Gcg*	.0116	3.02	TC0200003628.mm.2
*L1cam*	.0003	3.03	TC0X00003429.mm.2
*Suox*	.0005	3.05	TC1000003158.mm.2
*Sval1*	.0001	3.14	TC0600000448.mm.2
*Gm4788*	.0013	3.16	TC0100003190.mm.2
*Lrmp*	.0109	3.17	TC0600001709.mm.2
*Spink4*	.0219	3.18	TC0400000351.mm.2
*Rad51*	.0329	3.25	TC0200001760.mm.2
*Hsph1*	.0055	3.26	TC0500003660.mm.2
*Trpm6*	.0003	3.27	TC1900000327.mm.2
*Fam3b*	.0099	3.28	TC1600002150.mm.2
*Frmd3*	.0375	3.31	TC0400000774.mm.2
*Sis*	.0226	3.32	TC0300002151.mm.2
*Reg3a*	.0004	3.34	TC0600000876.mm.2
*Maoa*	.0016	3.41	TC0X00000181.mm.2
*Kcnk6*	.0081	3.59	TC0700002718.mm.2
*Erdr1*	.003	3.6	TC0Y00000223.mm.2
*Slc13a1*	9.12E-06	3.7	TC0600001939.mm.2
*Slc10a2*	9.23E-05	3.74	TC0800001643.mm.2
*Fabp6*	1.86E-05	3.86	TC1100002570.mm.2
*Gm11437*	.0014	4.12	TC1100003409.mm.2
*Rasa4; Mir7035*	.0045	4.17	TC0500001597.mm.2
*Bcas1*	.0075	4.33	TC0200005249.mm.2
*Slc14a1*	.0003	4.52	TC1800001637.mm.2
*Dclk1*	.001	5.39	TC0300000419.mm.2
*Trpm5*	.0008	5.75	TC0700004579.mm.2
*Gm21887*	.0016	5.98	TC0X00001715.mm.2
*Dpep1*	.0038	7.06	TC0800001511.mm.2
*Hck*	.001	7.59	TC0200002308.mm.2
*Rgs13*	.0001	8.42	TC0100003205.mm.2
*Plb1*	1.06E-05	16.66	TC0500000337.mm.2
*Pmp22*	6.13E-05	23.97	TC1100000822.mm.2

## References

[1] Hsia Y., Sharland M., Jackson C., Wong I.C.K., Magrini N., Bielicki J.A. (2019). Consumption of oral antibiotic formulations for young children according to the WHO Access, Watch, Reserve (AWaRe) antibiotic groups: an analysis of sales data from 70 middle-income and high-income countries. Lancet Infect Dis.

[2] Fink G., D'Acremont V., Leslie H.H., Cohen J. (2020). Antibiotic exposure among children younger than 5 years in low-income and middle-income countries: a cross-sectional study of nationally representative facility-based and household-based surveys. Lancet Infect Dis.

[3] Krzyzaniak N., Pawlowska I., Bajorek B. (2016). Review of drug utilization patterns in NICUs worldwide. J Clin Pharm Ther.

[4] WHO (2017). WHO model list of essential medicines for children (6th list).

[5] Mukhopadhyay S., Sengupta S., Puopolo K.M. (2019). Challenges and opportunities for antibiotic stewardship among preterm infants. Arch Dis Child Fetal Neonatal.

[6] Sturkenboom M.C., Verhamme K.M., Nicolosi A., Murray M.L., Neubert A., Caudri D., Picelli G., Sen E.F., Giaquinto C., Cantarutti L., Baiardi P., Felisi M.G., Ceci A., Wong I.C., TEDDY European Network of Excellence (2008). Drug use in children: cohort study in three European countries. BMJ.

[7] Vaz L.E., Kleinman K.P., Raebel M.A., Nordin J.D., Lakoma M.D., Dutta-Linn M.M., Finkelstein J.A. (2014). Recent trends in outpatient antibiotic use in children. Pediatrics.

[8] Liem T.B., Krediet T.G., Fleer A., Egberts T.C., Rademaker C.M. (2010). Variation in antibiotic use in neonatal intensive care units in the Netherlands. J Antimicrob Chemother.

[9] Neubert A., Lukas K., Leis T., Dormann H., Brune K., Rascher W. (2010). Drug utilisation on a preterm and neonatal intensive care unit in Germany: a prospective, cohort-based analysis. Eur J Clin Pharmacol.

[10] Suryawanshi S., Pandit V., Suryawanshi P., Panditrao A. (2015). Antibiotic prescribing pattern in a tertiary level neonatal intensive care unit. J Clin Diagn Res.

[11] Murphy C., Nair J., Wrotniak B., Polischuk E., Islam S. (2020). Antibiotic treatments and patient outcomes in necrotizing enterocolitis. Am J Perinatol.

[12] Tang B.H., Wu Y.E., Kou C., Qi Y.J., Qi H., Xu H.Y., Leroux S., Huang X., Zhou Y., Zheng Y., Jacqz-Aigrain E., Shen A.D., Zhao W. (2019). Population pharmacokinetics and dosing optimization of amoxicillin in neonates and young infants. Antimicrob Agents Chemother.

[13] Wu Y.E., Wang Y.K., Tang B.H., Dong L., Li X., Zhang W., Li D.F., Tian L.Y., van den Anker J., You D.P., Zhao W. (2021). Population pharmacokinetics and dosing optimization of amoxicillin in Chinese infants. J Clin Pharmacol.

[14] van Donge T., Fuchs A., Leroux S., Pfister M., Rodieux F., Atkinson A., Giannoni E., van den Anker J., Bielicki J. (2020). Amoxicillin dosing regimens for the treatment of neonatal sepsis: balancing efficacy and neurotoxicity. Neonatology.

[15] Jones N.L., Koletzko S., Goodman K., Bontems P., Cadranel S., Casswall T., Czinn S., Gold B.D., Guarner J., Elitsur Y., Homan M., Kalach N., Kori M., Madrazo A., Megraud F., Papadopoulou A., Rowland M., Espghan N. (2017). Joint ESPGHAN/NASPGHAN guidelines for the management of Helicobacter pylori in children and adolescents (update 2016). J Pediatr Gastroenterol Nutr.

[16] Standing J.F., Ongas M.O., Ogwang C., Kagwanja N., Murunga S., Mwaringa S., Ali R., Mturi N., Timbwa M., Manyasi C., Mwalekwa L., Bandika V.L., Ogutu B., Waichungo J., Kipper K., Berkley J.A., Group F.-P.S. (2018). Dosing of ceftriaxone and metronidazole for children with severe acute malnutrition. Clin Pharmacol Ther.

[17] Commander S.J., Gao J., Zinkhan E.K., Heresi G., Courtney S.E., Lavery A.P., Delmore P., Sokol G.M., Moya F., Benjamin D., Bumpass T.G., Debski J., Erinjeri J., Sharma G., Tracy E.T., Smith P.B., Cohen-Wolkowiez M., Hornik C.P. (2020). Best Pharmaceuticals for Children Act-Pediatric Trials Network Steering Committee. Safety of metronidazole in late pre-term and term infants with complicated intra-abdominal infections. Pediatr Infect Dis J.

[18] Brook I. (2002). Clinical review: bacteremia caused by anaerobic bacteria in children. Crit Care.

[19] Sosnin N., Curtis N., Cranswick N., Chiletti R., Gwee A. (2019). Vancomycin is commonly under-dosed in critically ill children and neonates. Br J Clin Pharmacol.

[20] Sridharan K., Al-Daylami A., Ajjawi R., Ajooz H.A.A. (2019). Vancomycin use in a paediatric intensive care unit of a tertiary care hospital. Paediatr Drugs.

[21] Gupta A., Cifu A.S., Khanna S. (2018). Diagnosis and treatment of Clostridium difficile infection. JAMA.

[22] Alvarez A.M., Rathore M.H. (2019). Clostridium difficile infection in children. Adv Pediatr.

[23] Alexander V.N., Northrup V., Bizzarro M.J. (2011). Antibiotic exposure in the newborn intensive care unit and the risk of necrotizing enterocolitis. J Pediatr.

[24] Cotten C.M., Taylor S., Stoll B., Goldberg R.N., Hansen N.I., Sanchez P.J., Ambalavanan N., Benjamin D.K., NICHD Neonatl Research Network (2009). Prolonged duration of initial empirical antibiotic treatment is associated with increased rates of necrotizing enterocolitis and death for extremely low birth weight infants. Pediatrics.

[25] Esaiassen E., Fjalstad J.W., Juvet L.K., van den Anker J.N., Klingenberg C. (2017). Antibiotic exposure in neonates and early adverse outcomes: a systematic review and meta-analysis. J Antimicrob Chemother.

[26] Oosterloo B.C., van Elburg R.M., Rutten N.B., Bunkers C.M., Crijns C.E., Meijssen C.B., Oudshoorn J.H., Rijkers G.T., van der Ent C.K., Vlieger A.M. (2018). Wheezing and infantile colic are associated with neonatal antibiotic treatment. Pediatr Allergy Immunol.

[27] Leppalehto E., Partty A., Kalliomaki M., Loyttyniemi E., Isolauri E., Rautava S. (2018). Maternal intrapartum antibiotic administration and infantile colic: is there a connection?. Neonatology.

[28] Ahmadizar F., Vijverberg S.J.H., Arets H.G.M., de Boer A., Lang J.E., Garssen J., Kraneveld A., Maitland-van der Zee A.H. (2018). Early-life antibiotic exposure increases the risk of developing allergic symptoms later in life: a meta-analysis. Allergy.

[29] Obiakor C.V., Tun H.M., Bridgman S.L., Arrieta M.C., Kozyrskyj A.L. (2018). The association between early life antibiotic use and allergic disease in young children: recent insights and their implications. Expert Rev Clin Immunol.

[30] Kamphorst K., Vlieger A.M., Oosterloo B.C., Waarlo S., van Elburg R.M. (2021). Higher risk of allergies at 4-6 years of age after systemic antibiotics in the first week of life. Allergy.

[31] Ajslev T.A., Andersen C.S., Gamborg M., Sorensen T.I., Jess T. (2011). Childhood overweight after establishment of the gut microbiota: the role of delivery mode, pre-pregnancy weight and early administration of antibiotics. Int J Obes (Lond).

[32] Mbakwa C.A., Scheres L., Penders J., Mommers M., Thijs C., Arts I.C. (2016). Early life antibiotic exposure and weight development in children. J Pediatr.

[33] Murphy R., Stewart A.W., Braithwaite I., Beasley R., Hancox R.J., Mitchell E.A., ISAAC Phase Three Study Group (2014). Antibiotic treatment during infancy and increased body mass index in boys: an international cross-sectional study. Int J Obes (Lond).

[34] Trasande L., Blustein J., Liu M., Corwin E., Cox L.M., Blaser M.J. (2013). Infant antibiotic exposures and early-life body mass. Int J Obes (Lond).

[35] Cho I., Yamanishi S., Cox L., Methe B.A., Zavadil J., Li K., Gao Z., Mahana D., Raju K., Teitler I., Li H., Alekseyenko A.V., Blaser M.J. (2012). Antibiotics in early life alter the murine colonic microbiome and adiposity. Nature.

[36] Ley D., Desseyn J.L., Mischke M., Knol J., Turck D., Gottrand F. (2017). Early-life origin of intestinal inflammatory disorders. Nutr Rev.

[37] Ozkul C., Ruiz V.E., Battaglia T., Xu J., Roubaud-Baudron C., Cadwell K., Perez-Perez G.I., Blaser M.J. (2020). A single early-in-life antibiotic course increases susceptibility to DSS-induced colitis. Genome Med.

[38] Kamphorst K., Van Daele E., Vlieger A.M., Daams J.G., Knol J., van Elburg R.M. (2021). Early life antibiotics and childhood gastrointestinal disorders: a systematic review. BMJ Paediatr Open.

[39] Lee Y.S., Kim T.Y., Kim Y., Lee S.H., Kim S., Kang S.W., Yang J.Y., Baek I.J., Sung Y.H., Park Y.Y., Hwang S.W., O E., Kim K.S., Liu S., Kamada N., Gao N., Kweon M.N. (2018). Microbiota-derived lactate accelerates intestinal stem-cell-mediated epithelial development. Cell Host Microbe.

[40] Knoop K.A., McDonald K.G., Kulkarni D.H., Newberry R.D. (2016). Antibiotics promote inflammation through the translocation of native commensal colonic bacteria. Gut.

[41] Knoop K.A., Gustafsson J.K., McDonald K.G., Kulkarni D.H., Kassel R., Newberry R.D. (2017). Antibiotics promote the sampling of luminal antigens and bacteria via colonic goblet cell associated antigen passages. Gut Microbes.

[42] Cox L.M., Yamanishi S., Sohn J., Alekseyenko A.V., Leung J.M., Cho I., Kim S.G., Li H., Gao Z., Mahana D., Zarate Rodriguez J.G., Rogers A.B., Robine N., Loke P., Blaser M.J. (2014). Altering the intestinal microbiota during a critical developmental window has lasting metabolic consequences. Cell.

[43] Schumann A., Nutten S., Donnicola D., Comelli E.M., Mansourian R., Cherbut C., Corthesy-Theulaz I., Garcia-Rodenas C. (2005). Neonatal antibiotic treatment alters gastrointestinal tract developmental gene expression and intestinal barrier transcriptome. Physiol Genomics.

[44] Yoon H., Schaubeck M., Lagkouvardos I., Blesl A., Heinzlmeir S., Hahne H., Clavel T., Panda S., Ludwig C., Kuster B., Manichanh C., Kump P., Haller D., Hormannsperger G. (2018). Increased pancreatic protease activity in response to antibiotics impairs gut barrier and triggers colitis. Cell Mol Gastroenterol Hepatol.

[45] Wlodarska M., Willing B., Keeney K.M., Menendez A., Bergstrom K.S., Gill N., Russell S.L., Vallance B.A., Finlay B.B. (2011). Antibiotic treatment alters the colonic mucus layer and predisposes the host to exacerbated Citrobacter rodentium-induced colitis. Infect Immun.

[46] Zarrinpar A., Chaix A., Xu Z.Z., Chang M.W., Marotz C.A., Saghatelian A., Knight R., Panda S. (2018). Antibiotic-induced microbiome depletion alters metabolic homeostasis by affecting gut signaling and colonic metabolism. Nat Commun.

[47] Zhang X.S., Li J., Krautkramer K.A., Badri M., Battaglia T., Borbet T.C., Koh H., Ng S., Sibley R.A., Li Y., Pathmasiri W., Jindal S., Shields-Cutler R.R., Hillmann B., Al-Ghalith G.A., Ruiz V.E., Livanos A., van 't Wout A.B., Nagalingam N., Rogers A.B., Sumner S.J., Knights D., Denu J.M., Li H., Ruggles K.V., Bonneau R., Williamson R.A., Rauch M., Blaser M.J. (2018). Antibiotic-induced acceleration of type 1 diabetes alters maturation of innate intestinal immunity. Elife.

[48] Al Nabhani Z., Dulauroy S., Marques R., Cousu C., Al Bounny S., Dejardin F., Sparwasser T., Berard M., Cerf-Bensussan N., Eberl G. (2019). A weaning reaction to microbiota is required for resistance to immunopathologies in the adult. Immunity.

[49] Miyoshi J., Bobe A.M., Miyoshi S., Huang Y., Hubert N., Delmont T.O., Eren A.M., Leone V., Chang E.B. (2017). Peripartum antibiotics promote gut dysbiosis, loss of immune tolerance, and inflammatory bowel disease in genetically prone offspring. Cell Rep.

[50] Oosterloo B.C., Van't Land B., de Jager W., Rutten N.B., Klopping M., Garssen J., Vlieger A.M., van Elburg R.M. (2019). Neonatal antibiotic treatment is associated with an altered circulating immune marker profile at 1 year of age. Front Immunol.

[51] Olszak T., An D., Zeissig S., Vera M.P., Richter J., Franke A., Glickman J.N., Siebert R., Baron R.M., Kasper D.L., Blumberg R.S. (2012). Microbial exposure during early life has persistent effects on natural killer T cell function. Science.

[52] Scheer S., Medina T.S., Murison A., Taves M.D., Antignano F., Chenery A., Soma K.K., Perona-Wright G., Lupien M., Arrowsmith C.H., De Carvalho D.D., Zaph C. (2017). Early-life antibiotic treatment enhances the pathogenicity of CD4(+) T cells during intestinal inflammation. J Leukoc Biol.

[53] Knoop K.A., Gustafsson J.K., McDonald K.G., Kulkarni D.H., Coughlin P.E., McCrate S., Kim D., Hsieh C.S., Hogan S.P., Elson C.O., Tarr P.I., Newberry R.D. (2017). Microbial antigen encounter during a preweaning interval is critical for tolerance to gut bacteria. Sci Immunol.

[54] Candon S., Perez-Arroyo A., Marquet C., Valette F., Foray A.P., Pelletier B., Milani C., Ventura M., Bach J.F., Chatenoud L. (2015). Antibiotics in early life alter the gut microbiome and increase disease incidence in a spontaneous mouse model of autoimmune insulin-dependent diabetes. PLoS One.

[55] Gokulan K., Cerniglia C.E., Thomas C., Pineiro S.A., Khare S. (2017). Effects of residual levels of tetracycline on the barrier functions of human intestinal epithelial cells. Food Chem Toxicol.

[56] Kalghatgi S., Spina C.S., Costello J.C., Liesa M., Morones-Ramirez J.R., Slomovic S., Molina A., Shirihai O.S., Collins J.J. (2013). Bactericidal antibiotics induce mitochondrial dysfunction and oxidative damage in Mammalian cells. Sci Transl Med.

[57] Morgun A., Dzutsev A., Dong X., Greer R.L., Sexton D.J., Ravel J., Schuster M., Hsiao W., Matzinger P., Shulzhenko N. (2015). Uncovering effects of antibiotics on the host and microbiota using transkingdom gene networks. Gut.

[58] Morikawa K., Watabe H., Araake M., Morikawa S. (1996). Modulatory effect of antibiotics on cytokine production by human monocytes in vitro. Antimicrob Agents Chemother.

[59] Pious D.A., Hawley P. (1972). Effect of antibiotics on respiration in human cells. Pediatr Res.

[60] Auricchio S., Rubino A., Muerset G. (1965). Intestinal glycosidase activities in the human embryo, fetus, and newborn. Pediatrics.

[61] Henning S.J. (1981). Postnatal development: coordination of feeding, digestion, and metabolism. Am J Physiol.

[62] Van Beers E.H., Buller H.A., Grand R.J., Einerhand A.W., Dekker J. (1995). Intestinal brush border glycohydrolases: structure, function, and development. Crit Rev Biochem Mol Biol.

[63] Spence J.R., Lauf R., Shroyer N.F. (2011). Vertebrate intestinal endoderm development. Dev Dyn.

[64] Clark S.L. (1959). The ingestion of proteins and colloidal materials by columnar absorptive cells of the small intestine in suckling rats and mice. J Biophys Biochem Cytol.

[65] Rodewald R. (1973). Intestinal transport of antibodies in the newborn rat. J Cell Biol.

[66] Hatae T., Fujita M., Okuyama K. (1988). Study on the origin of apical tubules in ileal absorptive cells of suckling rats using concanavalin-A as a membrane-bound tracer. Cell Tissue Res.

[67] Muncan V., Heijmans J., Krasinski S.D., Buller N.V., Wildenberg M.E., Meisner S., Radonjic M., Stapleton K.A., Lamers W.H., Biemond I., van den Bergh Weerman M.A., O'Carroll D., Hardwick J.C., Hommes D.W., van den Brink G.R. (2011). Blimp1 regulates the transition of neonatal to adult intestinal epithelium. Nat Commun.

[68] Fujita M., Baba R., Shimamoto M., Sakuma Y., Fujimoto S. (2007). Molecular morphology of the digestive tract; macromolecules and food allergens are transferred intact across the intestinal absorptive cells during the neonatal-suckling period. Med Mol Morphol.

[69] Pacha J. (2000). Development of intestinal transport function in mammals. Physiol Rev.

[70] Skrzypek T.S., Ferenc S.K., Kazimierczak W., Szczepaniak K., Zabielski R. (2018). The contribution of vacuolated foetal-type enterocytes in the process of maturation of the small intestine in piglets. J Animal Feed Sci.

[71] Rath T., Kuo T.T., Baker K., Qiao S.W., Kobayashi K., Yoshida M., Roopenian D., Fiebiger E., Lencer W.I., Blumberg R.S. (2013). The immunologic functions of the neonatal Fc receptor for IgG. J Clin Immunol.

[72] Arevalo Sureda E., Westrom B., Pierzynowski S.G., Prykhodko O. (2016). Maturation of the intestinal epithelial barrier in neonatal rats coincides with decreased FcRn expression, replacement of vacuolated enterocytes and changed Blimp-1 expression. PLoS One.

[73] Hurwitz R., Kretchmer N. (1986). Development of arginine-synthesizing enzymes in mouse intestine. Am J Physiol.

[74] Krasinski S.D., Estrada G., Yeh K.Y., Yeh M., Traber P.G., Rings E.H., Buller H.A., Verhave M., Montgomery R.K., Grand R.J. (1994). Transcriptional regulation of intestinal hydrolase biosynthesis during postnatal development in rats. Am J Physiol.

[75] De Jonge W.J., Dingemanse M.A., de Boer P.A., Lamers W.H., Moorman A.F. (1998). Arginine-metabolizing enzymes in the developing rat small intestine. Pediatr Res.

[76] Menard S., Forster V., Lotz M., Gutle D., Duerr C.U., Gallo R.L., Henriques-Normark B., Putsep K., Andersson M., Glocker E.O., Hornef M.W. (2008). Developmental switch of intestinal antimicrobial peptide expression. J Exp Med.

[77] van Es J.H., Jay P., Gregorieff A., van Gijn M.E., Jonkheer S., Hatzis P., Thiele A., van den Born M., Begthel H., Brabletz T., Taketo M.M., Clevers H. (2005). Wnt signalling induces maturation of Paneth cells in intestinal crypts. Nat Cell Biol.

[78] Darmoul D., Brown D., Selsted M.E., Ouellette A.J. (1997). Cryptdin gene expression in developing mouse small intestine. Am J Physiol.

[79] Bry L., Falk P., Huttner K., Ouellette A., Midtvedt T., Gordon J.I. (1994). Paneth cell differentiation in the developing intestine of normal and transgenic mice. Proc Natl Acad Sci U S A.

[80] Navis M., Martins Garcia T., Renes I.B., Vermeulen J.L., Meisner S., Wildenberg M.E., van den Brink G.R., van Elburg R.M., Muncan V. (2019). Mouse fetal intestinal organoids: new model to study epithelial maturation from suckling to weaning. EMBO Rep.

[81] Garcia T.M., Navis M., Wildenberg M.E., van Elburg R.M., Muncan V. (2019). Recapitulating suckling-to-weaning transition in vitro using fetal intestinal organoids. J Vis Exp.

[82] Stanford A.H., Gong H., Noonan M., Lewis A.N., Gong Q., Lanik W.E., Hsieh J.J., Lueschow S.R., Frey M.R., Good M., McElroy S.J. (2020). A direct comparison of mouse and human intestinal development using epithelial gene expression patterns. Pediatr Res.

[83] Gasparrini A.J., Crofts T.S., Gibson M.K., Tarr P.I., Warner B.B., Dantas G. (2016). Antibiotic perturbation of the preterm infant gut microbiome and resistome. Gut Microbes.

[84] Shaw-Smith C.J., Walters J.R. (1997). Regional expression of intestinal genes for nutrient absorption. Gut.

[85] Anderle P., Sengstag T., Mutch D.M., Rumbo M., Praz V., Mansourian R., Delorenzi M., Williamson G., Roberts M.A. (2005). Changes in the transcriptional profile of transporters in the intestine along the anterior-posterior and crypt-villus axes. BMC Genomics.

[86] Fang R., Olds L.C., Sibley E. (2006). Spatio-temporal patterns of intestine-specific transcription factor expression during postnatal mouse gut development. Gene Expr Patterns.

[87] Yu K., Mu C., Yang Y., Su Y., Zhu W. (2017). Segment-specific responses of intestinal epithelium transcriptome to in-feed antibiotics in pigs. Physiol Genomics.

[88] Haber A.L., Biton M., Rogel N., Herbst R.H., Shekhar K., Smillie C., Burgin G., Delorey T.M., Howitt M.R., Katz Y., Tirosh I., Beyaz S., Dionne D., Zhang M., Raychowdhury R., Garrett W.S., Rozenblatt-Rosen O., Shi H.N., Yilmaz O., Xavier R.J., Regev A. (2017). A single-cell survey of the small intestinal epithelium. Nature.

[89] Mustata R.C., Vasile G., Fernandez-Vallone V., Strollo S., Lefort A., Libert F., Monteyne D., Perez-Morga D., Vassart G., Garcia M.I. (2013). Identification of Lgr5-independent spheroid-generating progenitors of the mouse fetal intestinal epithelium. Cell Rep.

[90] Fordham R.P., Yui S., Hannan N.R., Soendergaard C., Madgwick A., Schweiger P.J., Nielsen O.H., Vallier L., Pedersen R.A., Nakamura T., Watanabe M., Jensen K.B. (2013). Transplantation of expanded fetal intestinal progenitors contributes to colon regeneration after injury. Cell Stem Cell.

[91] Rodriguez-Colman M.J., Schewe M., Meerlo M., Stigter E., Gerrits J., Pras-Raves M., Sacchetti A., Hornsveld M., Oost K.C., Snippert H.J., Verhoeven-Duif N., Fodde R., Burgering B.M. (2017). Interplay between metabolic identities in the intestinal crypt supports stem cell function. Nature.

[92] Zhao D., Cai C., Zheng Q., Jin S., Song D., Shen J., Ran Z. (2017). Vancomycin pre-treatment impairs tissue healing in experimental colitis: importance of innate lymphoid cells. Biochem Biophys Res Commun.

[93] Feng Y., Huang Y., Wang Y., Wang P., Song H., Wang F. (2019). Antibiotics induced intestinal tight junction barrier dysfunction is associated with microbiota dysbiosis, activated NLRP3 inflammasome and autophagy. PLoS One.

[94] Ekstrom G.M., Westrom B.R., Telemo E., Karlsson B.W. (1988). The uptake of fluorescein-conjugated dextran 70,000 by the small intestinal epithelium of the young rat and pig in relation to macromolecular transmission into the blood. J Dev Physiol.

[95] Kim S.W., Ehrman J., Ahn M.R., Kondo J., Lopez A.A.M., Oh Y.S., Kim X.H., Crawley S.W., Goldenring J.R., Tyska M.J., Rericha E.C., Lau K.S. (2017). Shear stress induces noncanonical autophagy in intestinal epithelial monolayers. Mol Biol Cell.

[96] Ono K., Satoh Y. (1981). Ultrastructural localization of acid phosphatase activity in the small intestinal absorptive cells of postnatal rats. Histochemistry.

[97] Esner M., Graifer D., Lleonart M.E., Lyakhovich A. (2017). Targeting cancer cells through antibiotics-induced mitochondrial dysfunction requires autophagy inhibition. Cancer Lett.

[98] Arimura Y., Yano T., Hirano M., Sakamoto Y., Egashira N., Oishi R. (2012). Mitochondrial superoxide production contributes to vancomycin-induced renal tubular cell apoptosis. Free Radic Biol Med.

[99] King D.W., Smith M.A. (2004). Proliferative responses observed following vancomycin treatment in renal proximal tubule epithelial cells. Toxicol In Vitro.

[100] Sakamoto Y., Yano T., Hanada Y., Takeshita A., Inagaki F., Masuda S., Matsunaga N., Koyanagi S., Ohdo S. (2017). Vancomycin induces reactive oxygen species-dependent apoptosis via mitochondrial cardiolipin peroxidation in renal tubular epithelial cells. Eur J Pharmacol.

[101] He G., Shankar R.A., Chzhan M., Samouilov A., Kuppusamy P., Zweier J.L. (1999). Noninvasive measurement of anatomic structure and intraluminal oxygenation in the gastrointestinal tract of living mice with spatial and spectral EPR imaging. Proc Natl Acad Sci U S A.

[102] Ginglen J.G., Butki N. (2021). Necrotizing enterocolitis.

[103] Van Lidth de Jeude J.F., Vermeulen J.L., Montenegro-Miranda P.S., Van den Brink G.R., Heijmans J. (2015). A protocol for lentiviral transduction and downstream analysis of intestinal organoids. J Vis Exp.

[104] Schmidlin A., Kalbacher H., Wiesinger H. (1997). Presence of argininosuccinate synthetase in glial cells as revealed by peptide-specific antisera. Biol Chem.

